# Parenting support to prevent overweight during regular well-child visits in 0-3 year old children (BBOFT+ program), a cluster randomized trial on the effectiveness on child BMI and health behaviors and parenting

**DOI:** 10.1371/journal.pone.0237564

**Published:** 2020-08-18

**Authors:** Eline Vlasblom, Amy van Grieken, Maaike Beltman, Monique P. L’Hoir, Hein Raat, Magda M. Boere-Boonekamp

**Affiliations:** 1 Department of Child Health, TNO, Leiden, The Netherlands; 2 Department of Public Health, Erasmus Medical Center, Rotterdam, The Netherlands; 3 Department of Human Nutrition and Health, Wageningen University & Research, Wageningen, The Netherlands; 4 Department of Public Health Services of North- and East-Gelderland (GGDNOG), Warnsveld, The Netherlands; 5 Department of Health Technology and Services Research, Technical Medical Centre, University of Twente, Enschede, The Netherlands; University of Ghana, GHANA

## Abstract

**Background:**

Prevention of overweight during early childhood seems promising.

**Objective:**

To evaluate the effectiveness of the parenting-based BBOFT+ overweight prevention program on child BMI, child health behavior and parenting behavior among 0–36 month old children. BBOFT+ is an acronym for the key healthy lifestyle behaviors that are targeted in the BBOFT+ intervention: breastfeeding (B), daily breakfast (B), daily going outdoors (O), limiting sweet beverages (in Dutch, F) and minimal TV or computer time (T), complemented with healthy sleep behavior and improvement of parenting skills (+).

**Methods:**

A cluster randomized controlled trial in newborn children visiting well-baby clinics, comparing the BBOFT+ intervention (N = 901) with care as usual (CAU) (N = 1094). In both groups, parents received regular well-child visits (±11 visits in the first 3 years). In the intervention group, care was supplemented with the BBOFT+ program, which focuses on improving parenting skills from birth onwards to increase healthy behavior. Questionnaires were filled in at child's age 2–4 weeks, 6, 14 and 36 months. In multivariate analyses we corrected for child’s birthweight, age, ethnic background, mother’s educational level and BMI.

**Results:**

No differences were found in weight status at 36 months between intervention and control group children. At 6 months, BBOFT+ parents reported their child drinking less sweet beverages than control parents (48% vs 54%;p = .027), and going outdoors daily with their child less often (57% vs 62%;p = .03). At 14 months, more BBOFT+ parents than control parents reported to have breastfed for six months or longer (32% vs 29%;p = .022). At 36 months, more BBOFT+ parents than control parents reported their child going outside daily (78% vs 72%;p = .011) and having less TV/computer time on week- (38% vs 46%;p = .001) and weekend days (48% vs 56%;p = .002). Also, BBOFT+ parents reported having more parental control than control parents (3.92 vs 3.89;p = .02). No significant differences were found for daily breakfast, sleep duration and parenting practices in adjusted analyses.

**Conclusion:**

The BBOFT+ overweight prevention program showed small improvements in parent-reported child health behaviors, compared to care as usual; no effect was observed on child BMI. The identified modifiable elements are potentially relevant for interventions that aim to prevent overweight.

## Introduction

Over the past decades, early childhood overweight and obesity prevalence rates have been increasing dramatically worldwide [1]. In some developed countries, a stabilization or even decrease of the prevalence in children above 3 years is observed [2–4]. A slight decrease has also been observed in Dutch children. Nonetheless, the last Dutch nationwide study from 2009 showed that 13% of boys and 15% of girls aged 2–21 years were overweight and that there is a sharp increase in overweight between the age of 2–7 [5]. Therefore, the sense of urgency to intervene at an early age to prevent overweight and obesity remains high. Overweight in early childhood is a major risk factor for becoming obese in later childhood [6] and overweight in childhood often tracks into adulthood [7]. Childhood obesity is associated with increased morbidity in adult life, e.g., diabetes, coronary heart diseases and various types of cancer, and can result in high lifetime costs of care [8,9].

In early life, fundamental development of food preferences takes place and eating, physical activity and sleep habits become settled [10]. Parents play a crucial role in the development of healthy lifestyle behaviors of their young children [11]. Healthy behavior in early childhood can be improved if parents receive guidance during this period to enhance their parenting skills specifically addressing their child's lifestyle behavior [12,13]. However, programs aiming to improve parenting skills to support overweight prevention in early childhood show mixed results [13–15].

In the Netherlands about 90% of preschool children visit Youth Health Care (YHC), which is offered nation-wide and free of charge to all families [16]. YHC-teams consisting of a doctor, a nurse and an assistant, provide preventive services to families with children (0–18 years) in well-child clinics and schools. The preventive care concentrates on monitoring the child’s growth and development and the early detection of health problems. Since 2004, usual care in The Netherlands includes that YHC professionals follow the Overweight prevention protocol, a clinical guideline that aims: 1) to detect children with overweight, using international cut-offs and a set of physical criteria, and 2) to support their parents through up to three additional counseling visits [17].

In a study into the overweight prevention protocol only small effects on BMI were detected and improving the protocol itself and implementation of the protocol was recommended [18]. In order to equip professionals with skills to improve implementation of the protocol and to support parents to increase their child’s healthier lifestyle behaviors, the BBOFT+ overweight prevention program for parents of 0–3 year old children was developed. BBOFT+ is an acronym for the key healthy lifestyle behaviors that are targeted in the BBOFT+ intervention: breastfeeding (B), daily breakfast (B), daily going outdoors (O), limiting sweet beverages (in Dutch, F) and minimal TV or computer time (T) [17], complemented with healthy sleep behavior and improvement of parenting skills (+).

BBOFT+ was based on the YHC Overweight prevention protocol and results of a cross-sectional Dutch study that showed that relatively often, parents of 0–4 year old children held views and stimulated behavior that favored an unhealthy lifestyle of their child [19,20]. For example, in the study of Boere Boonekamp et al., [18] 40% of children always had soft drinks at their disposal, one in ten children aged 2–4 years old had a TV in their own bedroom, and one in seven children were not used to regular breakfast eating. The goal of the BBOFT+ intervention is to improve parenting skills by providing parents with education on basic principles of child rearing, and guidance by YHC-professionals on healthy lifestyle behavior during the regular well-child visits from birth until three years of age. The aim of this study was to assess the effects of the BBOFT+ intervention compared to usual care on child Body Mass Index (BMI), child health-related behavior, and parenting behavior (i.e. parenting styles and parenting practices), at child age 6, 14 and 36 months.

## Methods

### Study design

The BeeBOFT study is a cluster randomized controlled trial, with two intervention groups and one control group; the ‘BBOFT+’ intervention, the ‘E-health4Uth Healthy toddler’ intervention and the ‘Care as usual’ (CAU) groups. While the hypothesized effects on outcomes of both interventions were similar (e.g. both use BMI-SDS as a primary outcome) there were also differences in expected outcomes. Positive effects of the BBOFT+ intervention were expected on sleep and parenting outcomes, while these were not expected of the E-health4Uth Healthy toddler intervention. Therefore, both interventions were analyzed and published separately. The trial protocol [21] and the evaluation of the ‘E-health4Uth Healthy toddler’ [22] have been described elsewhere. In this paper we describe the evaluation of the BBOFT+ intervention compared to CAU, following the CONSORT guidelines, extended to cluster randomized trials [23].

### Participants and procedure

Ten YHC organizations participated (name, region): Zorggroep Oude en Nieuwe Land, Steenwijk; Careyn, Spijkenisse; Rivas, Gorinchem; Yunio, Doetinchem; Zorgboog, Bakel; CJG Rijnmond, Rotterdam; Stromen op Maat, Zwijndrecht; Vivent, Vught; Thebe, Tilburg; GGD Twente, Almelo), with 51 YHC teams (three times seventeen teams). The YHC team, consisting of community physicians and nurses, was the unit of randomization. Data were collected between January 2009 and September 2013. Participants were parents and their children. Parents with a child born between January 2009 and September 2010 were informed about the study during the regular home visit of the YHC nurse in the second week after birth and invited to participate. Parents unable to read the Dutch language were excluded from participating in the study. Of 7985 invited parents (the total number of births in the coverage area of participating YHC teams during the study period), 3003 parents agreed to participate in the study and provided written informed consent (participation rate of 37.6%).

At inclusion, a baseline questionnaire with questions on background characteristics was completed; 3 participants did not return this questionnaire. When the child was 6, 14, and 36 months of age, parents were asked to complete an extensive questionnaire, on paper or online, containing items on primary and secondary outcomes. The questionnaires could be completed either by the mother or the father. Parents received a small gift that was sent with each questionnaire. The response rates at the three ages were 77.6% (2331/3003), 77.2% (2318/3003), and 73.5% (2206/3003), respectively.

### Intervention

The ‘BBOFT+’ intervention included targeted education and guidance of parents in applying the principles of stimulus control, modeling and classic conditioning, thereby increasing positive parenting skills, by YHC professionals (community physicians and nurses). The rationale of the intervention is that, by anticipating on common problems, it enables parents to create the conditions that stimulate the desired healthy behavior in the child by increasing children’s self-esteem, setting a good example, using praise and reward, managing children’s problem behaviors by setting ground rules, giving clear instructions and the use of consistent measures [21].

Parents in the ‘BBOFT+’ group received the intervention during all well-child visits, i.e. 8 to 13 visits of 10–20 minutes in the first three years. To support counseling, the YHC professionals used a small, calendar-like booklet that was placed on their desk. The front side of the booklet consisted of pictures of parents and children illustrating the desired behavior, the backside provided all age-appropriate items (8–15 per visit) to be discussed with parents by YHC professionals during the visits (Table 1). For example: “a baby and TV watching don’t match” until the age of 9 months, and “Watch TV/play on the computer together, no longer than 1 hour” from the age of 24 months. The booklet was specifically designed to be suited for all parents, including those with low literacy skills

**Table 1:** Schematic overview of the ‘BBOFT+’ intervention [21].

Feeding	• Breastfeeding	± 2 weeks
	• Variation in maternal food	± 1,2 months
	• No extra bottle when breastfeeding	± 2 weeks, ± 2 months
	• Leftovers allowed in bottle	± 2 weeks, ± 2 months
	• No extra supplements in bottle	± 1 month
	• Level spoon for bottle feeding	± 1 month
	• Do not reward every cry or fuss with feeding	± 2 months
	• Eat in a social setting (not in front of TV)	± 1 month
	• Accustom to different structures	± 3 months
	• Accustom to different tastes	± 4, 7½ months
	• Difference home-made food and food from jars	± 6 months
	• Eating at the same time at the table	± 4,6, 7½ months
	• No TV watching while eating	± 9,11,18,24,36 months
	• Positive atmosphere at the table	± 9,11,18,36 months
	• Child may eat less or more in this phase	± 14,24 months
Space to move and play with pleasure	• Tummy time under supervision	± 2 weeks, 1,2,3,4,6 months
	• Car seat is for transport	± 4 months
	• Not too long in rocking chair	± 4 months
	• Get baby out of playpen before it starts to cry	± 4 months
	• The playpen is safe and nice place to play	± 6, 7½, 11 months
	• The playpen is a stimulant for motoric development	± 6, 7½, 11 months
	• Let the toddler walk itself when/where possible	± 14,18,24,36 months
Daily outside		± 2 weeks, 1,2,3,6,9,11,14,18 months
Sleep	• Duration (sleeping/awake)	± 2 weeks, 1,2,3,4,6, 7½, 9,11, 18,24,36 months
	• Put the baby to sleep awake	± 2,3, 7½ months
	• Sleep in afternoon gradually reducing	±7½ months
	• Late feeding in the evening not necessary anymore	±7½,9,11,18 months
	• Children like rituals when going to bed	± 9,11,24,36 months
	• A bottle “to fall asleep” is not necessary	± 9,11,14 months
	• Set bedtime	± 11,18,24,36 months
Regularity, uniformity in daily care and reduction of stimuli	• Fixed order: sleeping, feeding, playing, getting tired, bring to bed awake	± 1,2,11,14,18 months
	• Crying increases till 6–8 weeks, and decreases after 8 weeks	± 1 months
	• Play in playpen, transport in car seat	± 2 months
Parenting	• Role/influence grandparents	± 2, 7½, 18,36 months
	• Children need warmth, love and safety	± 2,3, 7½ months
	• Sensitive and warm parenting and at the same time restriction	± 7½, 9,14,18,24,36 months
	• Sweets/food not to be used as reinforcer of behavior	± 7½,9,14,18 months
	• Authoritative parenting style	± 9,24 months
	• Children like predictability	± 14 months
	• Screen/food not to be used to reinforce of behavior	± 24,36 months
Screen time	• A baby and TV watching don’t match	± 3,4,6,7 1/2,9 months
	• No television in bedroom of the child	± 11,14,18,24,36 months
	• Watch TV together	± 14 months
	• Watch TV/play computer together, not longer than 1 hour, daily	± 24,36 months
Drinking	• When thirsty, offer water	± 4,6,9,11,18,24 months
Snack	• Water and diluted fruit juice or tea (no sugar), and bread crust or cracker	± 9,11 months

The intervention comprised of several steps: 1. Building a positive work relation with the parent; 2. Risk assessment; 3. Introducing the booklet; 4. Asking the parents which items of the booklet they would like to focus on; 5. Providing information about the chosen items, after asking for permission to do so. The risk assessment was both aimed at high risk groups for overweight (for example overweight of the parents, high birth weight, low socio-economic status, etc.) and the knowledge level, worries and parenting competencies of the parents. This approach fits in a regular consult. If large concerns were put forward by the parent, the YHC professional could choose to arrange a home visit. During the home visit the YHC professional either used motivational interviewing or mediation to support behavior change. All YHC professionals in the BBOFT+ intervention arm received the BBOFT+ manual and followed two training sessions. The first training session, before the start of the intervention, was a 4-hour training on healthy child rearing, aspects of social learning theory (stimulus control, self-regulation, sensitive and responsive parenting, setting clear boundaries, etc.) and communication techniques (providing anticipating information, motivational interviewing and mediation techniques). The second session was a refresher training after a year, to motivate the BBOFT+ intervention teams and to ensure compliance to the intervention protocol. Both training sessions were delivered by a psychotherapist (author MLH).

### Care as usual

Parents assigned to the control group received CAU, consisting of the regular well-child visits with standard information to stimulate healthy child development. This might include information on feeding, physical activity, sleep routine, etc. From the age of 2 years, if necessary, obese children were referred to the pediatrician, in accordance with the Dutch YHC Overweight prevention protocol [17].

CAU differs from the BBOFT+ intervention in terms of content (in BBOFT+ more topics discussed, more specific and more elaborate information provided during well-child visits), methods and materials used (i.e. BBOFT+ includes education on social learning theory, motivational interviewing, mediation techniques, booklet and intervention manual) and the systematic approach that characterizes BBOFT+ (described above). The professionals in the CAU group did not receive any specific training regarding overweight prevention, nor specific supporting materials.

### Background characteristics

Data on background characteristics was collected at baseline. Background characteristics (parent-reported) were: child’s sex (male/female), birthweight, ethnic background (Dutch/non-Dutch); pregnancy duration (days); parental age (years), parental BMI (kg/m2; classified as either normal weight (BMI <25.0) or overweight/obese (BMI ≥25.0)), and education level (low/middle/high); and family composition. Pregnancy duration was calculated using parent-reported due date and actual date of birth of the child. The parents’ and child’s ethnic background were defined in accordance with the definition established by Statistics Netherlands: a parent/child was classified as non-Dutch if one of his/her own parents was born outside the Netherlands [24]. Highest attained education level was categorized into: 1) low: elementary or lower levels of secondary education; 2) middle: higher levels of secondary education or intermediate vocational education; 3) high: higher vocational education and university education [25]. Family composition was categorized as 1) child is living with both parents or 2) child is living with a single parent.

### Primary and secondary outcomes

The primary outcome measures were child BMI and child health-related behaviors. The secondary outcome was parenting behavior, measured by general parenting styles and parenting practices (see S1 Table).

The child’s BMI was calculated based on the height and weight data collected from the YHC files. These anthropometric data were measured during each YHC visit by a YHC professional, using standardized protocols [17]. Children were classified as obese, overweight or normal weight based on international age- and gender-specific cutoff values [26]. The Body Mass Index Standard Deviation Score (BMI SDS) per age was calculated using the 1980 Dutch reference population [5].

Questions on the child’s health-related behavior were asked at the age of 6, 14 and 36 months, and concerned the previous 4 weeks (see S1 Table). All health-related behavior questions were adapted from Dutch questionnaires that were used in previous studies [18,27,28].

Parenting styles were assessed using the parental warmth scale, at the age of 14 and 36 months and the parental control scale at 36 months, as developed by Wake et al. [29]. The parental warmth scale consists of six items. Each item can be scored on a 5-point Likert scale (1 = “never” to 5 = “very often”). The average on these six items was calculated, with a higher score indicating higher parental warmth. Cronbach’s alpha for parental warmth was .80 at T2 and .84 at T3. The parental control scale consists of five items. Each item can be scored on a 5-point Likert scale (1 = “never” to 5 = “very often”). The average on these five items was calculated, with a higher score indicating higher parental control. Cronbach’s alpha for parental control was .62 at T3.

Parenting practices were assessed with the Parenting strategies for Eating and Activity Scale (PEAS) at age 36 months [30]. Five subscales were completed: limit setting (6 items), control (6 items), monitoring (7 items), discipline (5 items) and reinforcement (2 items). At 14 months the subscale reinforcement parenting practice was also used. Each item was scored on a 5-point Likert scale (1 = “never” to 5 = “always”). The average score per subscale was calculated, with a higher score indicating higher use of the corresponding parenting practice. Cronbach’s alpha for the subscales was .83 for limit setting, .31 for control, .83 for monitoring and .87 for discipline and .80 for reinforcement.

All child health behavior variables (except for the continuous variable ‘total sleep duration’) were transformed into dichotomous variables (yes/no), by combining response categories (see Tables 3 and 4), because of non-normal distributed residuals.

### Randomization and blinding

Cluster randomization was performed by randomly assigning YHC teams within an organization to one of the two intervention groups or the control group using a computerized random allocation generator. This means that within an organization, some YHC teams with YHC professionals were delivering one of the two interventions (BBOFT+ or E-health4Uth Healthy toddler), and other teams with other YHC professionals were delivering care as usual, i.e. the interventions and care as usual were not delivered by the same individuals. Families generally visit the same team, as teams are location based. Parents, YHC professionals and research assistants were not blinded to the experimental conditions.

### Sample size

Sample size was calculated at 1250 parents/children (in total for the 3 groups, after an expected response rate of 50% and dropout between baseline and follow-up of 30%), based on equal standard deviations (1.3) in the interventions and the control groups, an alpha of 0.05 (2-tailed) and a power of 0.80 of a two-sided t-test, application of a correction factor to account for the cluster design (0.10), and minimal detectable differences of 0.4 in primary and secondary outcomes [21].

### Statistical analyses

To compare the characteristics between the BBOFT+ group and the CAU group, we used either the Student’s T-test (for continuous variables) or Chi-square test (for categorical variables).

To assess the effect of the BBOFT+ intervention on primary and secondary outcomes at 6, 14 and 36 months, multilevel analyses were applied to allow for dependency between the individual measurements within the YHC teams [31,32]. Multilevel linear regression analyses were applied for continuous outcome variables and multilevel logistic regression analyses for dichotomous outcome variables. Each outcome variable was evaluated as a dependent variable in separate models, with the experimental group (i.e., BBOFT+ or CAU) as independent variable. For each outcome we present the results of two regression models. The first model includes a correction for cluster (i.e. YHC team; the intra-class correlation coefficient ranged from 0.001 to 0.01); the second model includes corrections for cluster and covariates. Models in which a correction for previous measurements was added, only minimally changed the results, and are therefore not presented. Covariates were potential confounding factors, determined by 1) an association with the outcome, and 2) an unequal distribution between the intervention and control groups at baseline [31,32]. The following variables were added to model 2 as covariates: child ethnic background (Dutch vs Non-Dutch), mother’s educational level, mother’s BMI SDS and child’s birthweight. In addition, the models were corrected for the age of the child at the time of the follow-up assessment.

For the non-response analysis, we used descriptive statistics to compare the socio-demographic background variables of parents/children who did not complete the study (n = 904) after the baseline assessment and those who completed (n = 1091) the study (provided data on all outcome variables).

The analyses were conducted on an intention to treat basis, considering a two-tailed p-value < .05 as statistically significant and using IBM SPSS Statistics version 25.

### Ethical permission

The research proposal was reviewed by the Medical Ethics Committee of the Erasmus University Medical Center. The Committee concluded that the Medical Research Involving Human Subjects Act (in Dutch: Wet medisch-wetenschappelijk onderzoek met mensen) did not apply to this research proposal. The Medical Ethics Committee therefore approved the execution of this study (proposal number MEC-2008-250, November 14^th^, 2018). The trial was registered in The Netherlands Trial Register (reference number NTR1831). The trial was registered on May 2009, which is four months after the trial had started. Registration was delayed due to the high workload for preparing our large trial. There are no ongoing or related trials for this intervention.

## Results

### Study population

**Fig 1:**
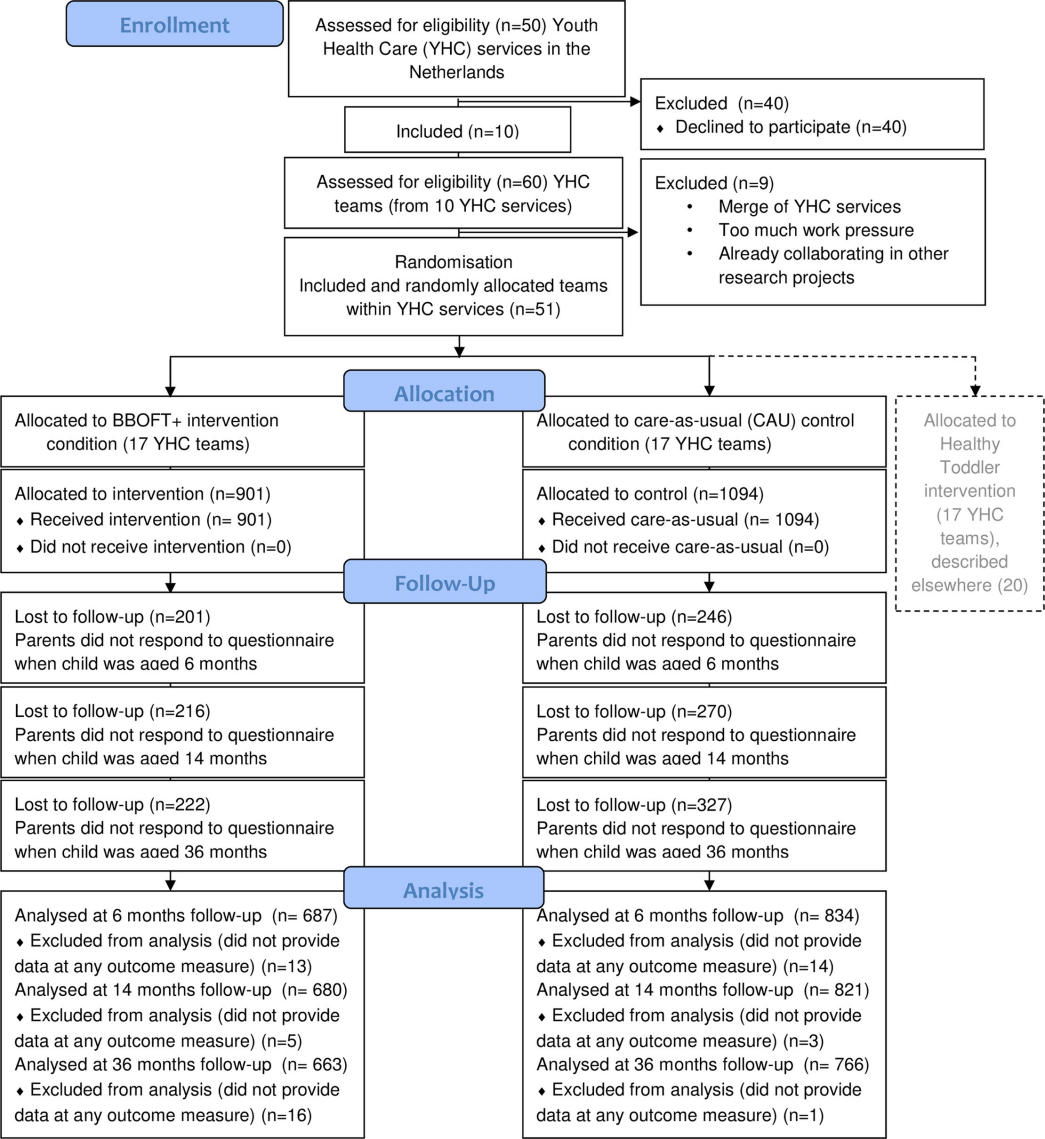
Flow diagram of participants in the study.

Fig 1 shows the flow of participants in the study. The YHC teams recruited 1094 parents for the CAU group and 901 parents for the BBOFT+ group. Mean child age at first, second and third assessment were 6, 14 and 36 months respectively. Table 2 shows the baseline characteristics of the children and their parents in both groups of the study sample. At baseline, there were significantly more mothers and fathers with a non-Dutch background (p = .004) and with a higher educational level (mothers: p = .014; fathers: p = .000) in the CAU group compared to the BBOFT+ group.

**Table 2:** Baseline characteristics of child, mother and father of the study sample (n = 1995).

	CAU	BBOFT+	p-value^1^
	n = 1094	n = 901	
**Child characteristics**			
Male gender (vs. female) (%) (missing n = 37)	567 (52.5%)	453 (51.5%)	.655
Birth weight in kilograms (mean, SD) (missing n = 11)	3453.6 (528.4)	3463.9 (558.5)	.673
Child is living with both parents (vs. single parent) (%) (missing n = 26)	1053 (97.7%)	874 (98.1%)	.530
Ethnic background (%) (missing n = 4)^2^			**< .001**
*Dutch*	862 (78.9%)	774 (86.1%)	
*Non-Dutch*	230 (21.1%)	125 (13.9%)	
**Characteristics of the mother**			
Pregnancy duration in days (mean, SD) (missing n = 76)	277.0 (11.0)	276.7 (12.0)	.526
Age in years at child’s birth (mean, SD) (missing n = 34)	31.0 (4.4)	30.8 (4.3)	.392
BMI kg/m2 (mean, SD) (missing n = 154)	25.3 (4.5)	25.3 (4.3)	.854
Ethnic background (%) (missing n = 4)			**.004**
*Dutch*	941 (86.2%)	821 (91.3%)	
*Non-Dutch*	151 (13.8%)	78 (8.7%)	
Education level (%) (missing n = 39)			**.014**
*Low*	148 (13.8%)	135 (15.3%)	
*Mid*	359 (33.4%)	339 (38.5%)	
*High*	568 (52.8%)	407 (46.2%)	
**Characteristics of the father**			
Age in years at child’s birth (mean, SD) (missing n = 89)	33.6 (5.1)	33.5 (5.0)	.943
BMI kg/m2 (mean, SD) (missing n = 224)	25.3 (3.1)	25.3 (3.6)	.740
Ethnic background (%) (missing n = 25)			**.004**
*Dutch*	931 (86.2%)	810 (91.1%)	
*Non-Dutch*	149 (13.8%)	80 (8.9%)	
Education level (%) (missing n = 111)			**< .001**
*Low*	211 (20.3%)	135 (16.0%)	
*Mid*	344 (33.1%)	356 (42.1%)	
*High*	484 (46.6%)	354 (41.9%)	

^1^P-value based on independent t-test for continuous variables and Chi-square test for categorical variables to analyze the difference between the control and intervention group.

^2^ Ethnic background of the child was based on the grandparents’ country of birth.

Bold numbers indicate *p <* .*05*.

### Primary outcomes

Table 3 presents the effects of the BBOFT+ intervention compared to CAU on child BMI SDS at the age of 6, 14 and 36 months. No significant differences were found regarding BMI SDS in the two groups at each of the three assessment points. At the age of 36 months, 6.1% of the children in the BBOFT+ group were classified as overweight or obese, and 4.0% of the children in the control group were overweight or obese.

**Table 3:** Intervention effects on child BMI SDS at child age 6, 14 and 36 months.

	CAU	BBOFT+	Model 1^a^	Model 2^b^
	Mean (SD)	Mean (SD)	B (95% CI)	B (95% CI)
Child age 6 months				
BMI SDS	0.21 (1)	0.29 (0.97)	0.088 (-0.032–0.208)	0.088 (-0.047–0.223)
Child age 14 months				
BMI SDS	-0.26 (0.95)	-0.22 (0.97)	0.042 (-0.095–0.179)	-0.012 (-0.12–0.097)
Child age 36 months				
BMI SDS	-0.15 (1.03)	-0.06 (1.08)	0.089 (-0.07–0.247)	0.048 (-0.124–0.22)

^a^Model 1: Adjusted for YHC team cluster

^b^Model 2: Adjusted for YHC team cluster, background characteristics (child ethnic background (Dutch vs Non-Dutch), mother’s educational level, mother’s BMI SDS, child’s birthweight) and child’s age at assessment.

Table 4 presents the results of the regression analysis, in which we evaluated the effects of the BBOFT+ intervention compared to CAU on child health behavior at each assessment point. At the age of 6 months, less children in the BBOFT+ group consumed sweet beverages (p = .027) and less children went outside daily (p = .030), compared to children in the CAU group. Without correcting for covariates, less children in the BBOFT+ group watched TV (p = .036) and children in the BBOFT+ group slept longer (p = .003) (in model 2 no longer significant).

**Table 4:** Intervention effects on child health behavior at child age 6, 14 and 36 months.

	CAU	BBOFT+	Model 1^a^	Model 2^b^
	Mean (SD) or % “yes” (n)	Mean (SD) or % “yes” (n)	OR/ B (95% CI)	OR/ B (95% CI)
**Child age 6 months**	**n = 834**	**n = 687**		
Drinking SB (vs. never drinking SB)	54.1% (448)	48.4% (331)	0.793 (0.647–0.972)*	0.777 (0.621–0.971)*
Going outside daily (vs.<7 days a week)	61.7% (513)	57.1% (392)	0.823 (0.67–1.012)	0.785 (0.63–0.977)*
Watching TV (vs. never TV watching)	52.6% (438)	47.2% (324)	0.805 (0.657–0.986)*	0.818 (0.658–1.017)
Total sleep duration in hours	14.8 (1.6)	15.0 (1.6)	0.181 (0.017–0.345)*	0.125 (-0.046–0.296)
**Child age 14 months**	**n = 821**	**n = 680**		
Drinking ≥3 SB per day (vs. <3 drinks) on weekdays	43.1% (347)	40.8% (273)	0.906 (0.736–1.116)	0.89 (0.713–1.111)
Drinking ≥3 SB per day (vs. <3 drinks) on weekend days	44.1% (354)	43.8% (291)	0.984 (0.8–1.211)	0.966 (0.775–1.205)
Going outside daily (vs.<7 days a week)	70.3% (572)	73.4% (496)	1.166 (0.929–1.464)	1.022 (0.765–1.366)
Watching TV (vs. never TV watching)	78.9% (642)	74.4% (503)	0.779 (0.612–0.992)*	0.785 (0.608–1.015)
Daily breakfast (vs.<7 days a week)	98% (802)	96.9% (658)	0.636 (0.329–1.23)	0.667 (0.318–1.398)
Breastfeeding for ≥6 months (vs. <6 months)	29.4% (239)	32.2% (218)	1.143 (0.916–1.426)	1.324 (1.041–1.685)*
Total sleep duration in hours	14.1 (1.36)	14.2 (1.4)	0.134 (-0.018–0.286)	0.109 (-0.033–0.251)
**Child age 36 months**	**n = 766**	**n = 663**		
Drinking ≥3 SB per day (vs. <3 drinks) on weekdays	36.7% (278)	37% (240)	1.013 (0.815–1.259)	0.882 (0.685–1.136)
Drinking ≥3 SB per day (vs. <3 drinks) on weekend days	39.7% (300)	41.1% (269)	1.062 (0.858–1.315)	0.982 (0.767–1.257)
Going outside daily (vs. <7 days a week)	72.4% (548)	78.3% (511)	1.372 (1.074–1.754)*	1.441 (1.088–1.910)*
TV and computer time ≥1 hour per day (vs. <1 hour) on weekdays	45.8% (350)	37.6% (247)	0.713 (0.576–0.882)**	0.652 (0.506–0.839)**
TV and computer time ≥1 hour per day (vs. <1 hour) on weekend days	56.3% (430)	48.2% (317)	0.724 (0.587–0.893)**	0.678 (0.531–0.866)**
Daily breakfast (vs. <7 days a week)	96.7% (739)	97.7% (643)	1.45 (0.758–2.776)	1.785 (0.817–3.899)
Total sleep duration in hours	12.0 (1.3)	12.0 (1.3)	0.008 (-0.128–0.144)	-0.121 (-0.278–0.037)

SB = sweet beverages

^a^Model 1: Adjusted for YHC team cluster

^b^Model 2: Adjusted for YHC team cluster, background characteristics (child ethnic background (Dutch vs Non-Dutch), mother’s educational level, mother’s BMI SDS, child’s birthweight) and child’s age at assessment.

*p < .*05*

**p < .*01*

At a child's age of 14 months, parents in the BBOFT+ group reported to have breastfed for six months or longer more often than parents in the CAU group (p = .022). Again, less children in the BBOFT+ group watched TV (p = .043) (in model 2 no longer significant).

At the age of 36 months, less children in the BBOFT+ group watched TV and/or used the computer for more than one hour a day compared to children in the CAU group: weekdays (p = .001), weekend days (p = .002). More children in the BBOFT+ group were going outside daily compared to the CAU group (p = .011).

### Secondary outcomes

Table 5 summarizes the results of the regression analysis in which we evaluated the effects of the BBOFT+ intervention compared to CAU on parenting behavior, measured by general parenting styles and parenting practices. Without adjusting for covariates, parents in the CAU group had a warmer parenting style than parents in the BBOFT+ group (p = .034) at child age 14 months (in model 2 no longer significant). At a child’s age of 36 months, the mean score on parental control for parents in the BBOFT+ group was significantly higher than for parents in the CAU group (3.92 versus 3.89, p = .020, after adjusting for covariates), indicating higher parental control in the intervention group. After adjusting for YHC cluster and covariates, no significant differences were found regarding warm parenting style and all five parenting practices between the BBOFT+ and the CAU group.

**Table 5:** Intervention effects on general parenting styles and parenting practices at child age 14 and 36 months.

	CAU	BBOFT+	Model 1^a^	Model 2^b^
	Mean (SD) or % “yes” (n)	Mean (SD) or % “yes” (n)	OR/ B (95% CI)	OR/ B (95% CI)
**Child age 14 months**	**n = 821**	**n = 680**		
Warm parenting style	4.38 (0.41)	4.33 (0.39)	-0.044 (-0.085 - -0.003)*	-0.041 (-0.084–0.002)
Reinforcement parenting practice	2.89 (1.17)	2.92 (1.17)	0.021 (-0.121–0.163)	0.014 (-0.111–0.14)
**Child age 36 months**	**n = 766**	**n = 663**		
Warm parenting style	4.47 (0.46)	4.47 (0.43)	-0.007 (-0.063–0.048)	0.018 (-0.031–0.067)
Control parenting style	3.89 (0.49)	3.92 (0.45)	0.03 (-0.02–0.079)	0.061 (0.006–0.117)*
Limit setting parenting practice	4.19 (0.86)	4.19 (0.88)	-0.001 (-0.092–0.09)	0.02 (-0.084–0.123)
Control parenting practice	2.74 (0.62)	2.72 (0.5)	-0.035 (-0.119–0.049)	-0.043 (-0.128–0.042)
Monitoring parenting practice	4.5 (0.51)	4.48 (0.53)	-0.017 (-0.079–0.046)	0.019 (-0.042–0.081)
Discipline parenting practice	3.79 (1.15)	3.91 (1.08)	0.116 (-0.001–0.234)	0.117 (-0.034–0.268)
Reïnforcement parenting practice	3.12 (1.14)	3.09 (1.17)	-0.023 (-0.194–0.149)	0.199 (0.011–0.387)

^a^Model 1: Adjusted for YHC team cluster

^b^Model 2: Adjusted for YHC team cluster, background characteristics (child ethnic background (Dutch vs Non-Dutch), mother’s educational level, mother’s BMI SDS, child’s birthweight) and child’s age at assessment.

*p < .*05*

### Non-response analysis

Parents who did not complete the study after the baseline assessment were more likely to be single parents (p = .007), of non-Dutch ethnicity (mothers p = .002, fathers p = .006), had a lower educational level (mothers p < .001, fathers p < .001), and were younger (mothers p < .001, fathers not significant), as compared to parents who completed the study. There were no significant differences between study completers and non-completers on any other baseline characteristic or intervention/control group (see S2 Table).

## Discussion

### Summary of results

The goal of our study was to evaluate the effects of the BBOFT+ intervention, including targeted education and guidance of parents by YHC professionals from a few weeks after childbirth onwards, on child BMI, health-related child behavior and parenting behavior. The aim of the education and guidance during well-child visits (child age 0–36 months) was to improve parenting skills and thereby creating the conditions for the desired behavior in the child. After adjustment for both YHC team cluster and confounders we found significant differences in some health-related behaviors between BBOFT+ and CAU children at all three assessment points: lower sweet beverages intake (6 months), longer breastfeeding (14 months), less daily outdoor activities (6 months), more daily outdoor activities (36 months), and less television/computer use (36 months). No differences were found regarding BMI SDS or weight status in the two groups at any of the three assessment points. In the total group, 4% of children were overweight (excluding obesity) and 1% were obese at age 36 months. Regarding secondary outcomes, parents in the BBOFT+ group scored higher on parental control compared to parents in the CAU group at age 36 months.

### Interpretation of the results

Our findings of small positive effects of the BBOFT+ intervention were consistent with several recent reviews and meta-analyses of overweight prevention programs which conclude that in this young age group small effects on children’s health-related behaviors can be achieved, while effects on BMI are sparse [13–15].

A possible explanation for not finding an effect of BBOFT+ on child BMI is that in the BBOFT+ study population the prevalence of overweight and obesity at 36 months proved to be rather low in both the intervention (6%) and CAU group (4%). This may have limited the potential effects that could have been observed in our trial. The prevalence rates in our study were also low in comparison with rates for overweight (including obesity) in the Netherlands in 2009 at the age of 3 years: 8% (boys) and 12% (girls) [5]. Our ‘healthier’ sample can be explained by the relatively higher number of parents with a high educational level (lower overweight prevalence) and the underrepresentation of parents with a non-Western background (higher overweight prevalence) [33–35]. These populations are known to be difficult to recruit for research participation, for example because there is mistrust in the research process, low literacy, or because daily life stressors make research participation not a priority [36]. Despite this, our intervention reached these families, as it was delivered to all parents visiting YHC and our materials were specifically designed to be suited for all parents, including those with low literacy skills. It is also possible that we have a ‘healthier’ sample because more parents had already become aware of the risks of overweight, induced by the greater focus of usual YHC care on prevention since 2004 [17]. This greater awareness is supported by a study from 2015 showing a slight nationwide reduction in overweight and obesity in very young children in The Netherlands [2]. However, the Dutch national steep increase in overweight prevalence between 2 and 7 years [5] and the still high prevalence of unhealthy lifestyle behaviors among the children in our sample justify the need for an intervention directed at children 0–3 years of age.

We found significant but small effects of our intervention on the child health-related behaviors sweet beverages intake, breastfeeding, daily outdoor activities and television/computer use, while we did not find significant effects on sleep duration and breakfast eating at any assessment time in our final models adjusted for cluster and confounding factors. This is in line with several other studies [13–15]. For example, a very similar intervention to the BBOFT+ intervention was evaluated by Schroeder et al., in the USA [37]. This intervention was also delivered by a healthcare provider at each well-baby visit (7–9 visits over 2 years), using the Growing Leaps and Bounds program. The intervention included verbal, visual and text advice and information for parents. There was a significant effect of the intervention on sweet beverage intake, and later introduction of food and drinks other than breastfeeding and no difference in BMI at 6, 12 and 24 months. Although Schroeder et al., did not find an effect of their intervention on parental control like we did, they did find that parents in the intervention group monitored and restricted their infant feeding more than parents in the control group.

There are several possible explanations for the small effects of our intervention on child health-related behaviors. First, we aimed to influence parenting behavior (styles and practices) in order to improve child health-related behaviors. To make parents conscious about the influence they have on their child’s behavior, our intervention started a few weeks after childbirth, before child health behaviors have become settled. However, according to our findings, none of the parenting behaviors were changed except for parental control. The increase of parental control in BBOFT+ parents was as hypothesized, because the BBOFT+ intervention promotes an authoritative parenting style. Parents with an authoritative parenting style have both a high level of sensitivity (“warmth” scale) and also a high level of expected self-control (“control” scale). In this case, parents give attention to the child’s own opinion and are emotionally involved, but simultaneously set clear boundaries [38]. Gerards et al. conclude in their review that interventions focusing on changing parenting styles could be most effective in young children. It is possible that increasing efforts in our intervention into changing parenting styles would have led to more positive intervention effects [39].

Another possible explanation for the small effects of our intervention on child health-related behaviors is that other factors are involved in behavior change, which were not the focus in our BBOFT+ intervention. For example, the behavior change technique “prompting generalization of a target behavior” was shown to be part of effective interventions for the prevention of obesity in children above 2 years old in a systematic review [40]. We did not include this behavior change technique explicitly in the BBOFT+ intervention. The behavior-change techniques “providing information on the consequences of behavior in general”, “providing rewards contingent on successful behavior” and “facilitating social comparison” were included in non-effective interventions, according to the review of Martin et al. [40]. Although not explicitly included in the BBOFT+ intervention, these might have been used by healthcare professionals in the BBOFT+ intervention.

A third potential explanation could be that two training sessions of health professionals may not have been sufficient to equip YHC community physicians and nurses with adequate skills to educate parents to increase their child’s healthy behavior. Our intervention might yield better results if the training for YHC professionals was more extensive and refreshers more frequent. A counter argument is however, that community physicians and nurses in standard Dutch YHC are already trained to tailor their advice to specific parental needs [16,17]. We did not assess implementation fidelity nor attendance rate of the well-child visits and therefore do not know to what extend parents received the BBOFT+ intervention or CAU.

There are several opportunities for improving our intervention which emerged from recent literature. In a systematic review of interventions aimed at reducing the risk of overweight and obesity during infancy and early childhood it was shown that nutritional or responsive feeding interventions targeted at parents of infants, improved feeding practices and had some impact on child weight, while parenting and family lifestyle interventions only improved some feeding practices, but not child weight [13]. Based on this review, our intervention might benefit from a greater focus on nutrition and responsive feeding. Also, some evidence suggests that parent support interventions for the prevention of overweight and obesity might be more effective if delivered through group education [41]. Adding E-health, as investigated in the other BBOFT intervention arm (E-health4Uth Healthy Toddler), specifically directed at tailoring healthy lifestyle advice, might increase the behavior change effect [22]. Blake-Lamb et al., suggests overweight prevention programs in early childhood should simultaneously address multiple obesity risk factors across several levels of influence and a variety of social sectors and be guided by an appropriate conceptual framework [15]. They suggest using the collective impact model for designing complex interventions for the prevention of obesity in early childhood [15]. Finally, our intervention might be more effective if it was not only applied in the setting of well-child visits, but also in settings such as home visits [15] and daycare facilities [42], and started earlier, during pregnancy [13].

### Strengths and limitations

The strengths of our study include the use of objectively assessed children’s weight and height and the robust cluster randomized controlled trial design. Because of the large sample size, our study was well powered. We were able to include a substantial number of parents with low educational level. This increases the generalizability of the results.

Another strength is that the study was conducted in the setting of daily YHC practice, which not only enabled us to perform a large-scale cluster RCT but also facilitates future implementation. Furthermore, the BBOFT+ intervention is low-intensive and relatively inexpensive, and therefore ideally suited to be implemented in well-child clinics of YHC. BBOFT+ enables healthcare professionals to improve parenting skills in all parents resulting in prevention of unhealthy child behaviors.

A limitation of our study is that despite cluster-randomization, there were baseline differences between the intervention and control group in educational level and ethnic background of parents. The BBOFT+ population was slightly lower educated and more often had a Dutch ethnic background than the CAU population. It is unknown if this difference is coincidental, or if it is caused by non-blind participation of parents in intervention or CAU group. Because of the difference between groups at baseline, we adjusted for background characteristics in the final model of our analysis. Another limitation of our study is that measures on child health-related behavior and parenting behavior came from parental reports, which are susceptible to social desirability bias [43]. We tried to limit this bias by ensuring anonymity for parents. We do not expect that the social desirability bias influenced the results of this study, as giving socially desirable answers—if present—probably was equal across both the intervention and control groups.

We did not adjust for multiple comparisons. If we would adjust for multiple comparisons, our results on going outside daily, TV watching and parental control in adjusted models at 36 months would remain statistically significant. The outcomes of our adjusted analyses on sweet beverages, going outside daily at 6 months, breastfeeding at 14 months and parental control at 36 months might have been found due to our large sample size and a higher risk of type 2 error [44].

We did not assess implementation fidelity during the well-child visits and therefore do not know if YHC professionals actually delivered the BBOFT+ intervention to all parents and, if they did, if it was delivered as intended [45]. The supporting materials and the refresher training aimed to increase implementation fidelity. Furthermore, we do not know if parents attended all well-child visits and if they were exposed to the intervention during all well-child visits. However, attendance rates to YHC in the Netherlands are generally high (above 90%) [16]. We have no reason to believe that the attendance rate during the BBOFT-study was different.

The effects of our intervention might become different as children age, when overweight becomes more prevalent [5]. However, it is unknow in which direction the effects would change, i.e., it is possible that effects might become (more) visible at a later age, or that our current small effects disappear over time. For this, a follow-up of our sample would be required.

## Conclusions

Overweight at a young age is related to numerous comorbidities and to an increased risk to become overweight as an adult. Our study found that children in the parenting support BBOFT+ overweight prevention program compared to usual care showed small improvements in parent-reported child health behaviors; no effect was observed on BMI. The BBOFT+ study identified some potentially modifiable elements for interventions that aim to prevent overweight and has potential to change the risk for overweight and obesity early in life.

## Supporting information

S1 Checklist CONSORT 2010 checklist of information to include when reporting a randomised trial*. (DOC)

S1 Table Summary of items for assessing background characteristics and health-related behaviors. (DOCX)

S2 Table Analyses of non-response. (DOCX)

S1 Data (PDF)
