# Supplementary material for: Parenting support to prevent overweight during regular well-child visits in 0-3 year old children (BBOFT+ program), a cluster randomized trial on the effectiveness on child BMI and health behaviors and parenting
Source: PLoS One. 2020 Aug 18;15(8):e0237564. doi: 10.1371/journal.pone.0237564 (PMC7437453; doi:10.1371/journal.pone.0237564)
Supplement: S1 Table — (DOCX) [file pone.0237564.s002.docx]

**S1 Table.** **Summary of items for assessing background characteristics and health-related behaviors.**

| Health behavior outcome | Questionnaire (age of the child in months) | Items | Response scale |
| --- | --- | --- | --- |
| Going outside daily | 14, 36 | How many days a week does your child go outside? | (almost) never, 1 day a week, 2 days a week, 3 days a week, 4 days a week, 5 days a week, 6 days a week, every day |
| Daily breakfast days/week | 14, 36 | How many days of the week does your child have breakfast? | Number of days: 0 to 7 |
| Sweet beverages | 6 | How often does your child consume on the following drinks:  fruit juices, carbonated soft drinks, noncarbonated soft drinks, lemonade syrup, milk and yoghurt drinks | Never, less than 1 time per week, 1-3 times per week, 4-6 times per week, 1-2 times per day, 3-4 times per day, more than 5 times per day |
| Sweet beverages on weekdays | 14, 36 | How many glasses of sweetened beverages does your child consume on average on a weekday ?  e.g. carbonated soft drinks, noncarbonated soft drinks, fruit juices, sport- and energy drinks, milk and yoghurt drinks, tea or water with sugar) | Less than 1, 1, 2, 3, 4, 5, 6, 7, 8, 9 or more |
| Sweet beverages on weekend days | 14, 36 | How many glasses of sweetened beverages does your child consume on average on a weekend day ?  e.g. carbonated soft drinks, noncarbonated soft drinks, fruit juices, sport- and energy drinks, milk and yoghurt drinks, tea or water with sugar) | Less than 1, 1, 2, 3, 4, 5, 6, 7, 8, 9 or more |
| TV viewing | 6 | Does your child watch TV? | Yes, no |
| TV viewing | 14 | How many days a week does your child on average watch television? | Less than 1 day per week, 1 day per week, 2 days per week, 3 days per week, 4 days per week, 5 days per week, 6 days per week, every day, not applicable (my child never watches TV) |
| TV viewing hours/ weekday | 36 | How much time per day does your child on average watch television on a week day? | Less than 30 minutes, 30 minutes to 1 hour, 1 to 2 hours, 2 to 3 hours, 3 to 4 hours, 4 to 5 hours, 5 to 6 hours, more than 6 hours, not applicable |
| TV viewing hours/ weekend day | 36 | How much time per day does your child on average watch television on a weekend day? | Less than 30 minutes, 30 minutes to 1 hour, 1 to 2 hours, 2 to 3 hours, 3 to 4 hours, 4 to 5 hours, 5 to 6 hours, more than 6 hours, not applicable |
| Computer use hours/ weekday | 36 | How much time per day does your child on average spent playing computer games on a week day? | Less than 30 minutes, 30 minutes to 1 hour, 1 to 2 hours, 2 to 3 hours, 3 to 4 hours, 4 to 5 hours, 5 to 6 hours, more than 6 hours, not applicable |
| Computer use hours/ weekend day | 36 | How much time per day does your child on average spent playing computer games on a weekend day? | Less than 30 minutes, 30 minutes to 1 hour, 1 to 2 hours, 2 to 3 hours, 3 to 4 hours, 4 to 5 hours, 5 to 6 hours, more than 6 hours, not applicable |
| Breastfeeding | 14 | How old was your child when the mother completely stopped breastfeeding? | Not applicable (the mother never started breastfeeding), the mother is still breastfeeding, before 6 months, between 6 and 7 months, between 7 and 8 months, between 8 and 9 months, between 9 and 10 months, between 10 and 11 months, between 11 and 12 months, longer than 12 months |
| Sleep duration | 6, 14 | Total sleep duration in hours during the night (between 18:00 and 8:00) | 8 hours or less, 9 hours, 10 hours, 11 hours, 12 hours, 13 hours, 14 hours or more |
| Sleep duration | 36 | Total sleep duration in hours during the night (between 18:00 and 8:00) | 7 hours or less, 8 hours, 9 hours, 10 hours, 11 hours, 12 hours, 13 hours, 14 hours or more |
| Sleep duration | 14, 36 | Total sleep duration in hours during the day (between 8:00 and 18:00) | 0 hours, 1 hour, 2 hours, 3 hours, 4 hours, 5 hours, 6 hours or more |
| Parenting practices | 14 (only subscale “reinforcement”)  36 | Parenting practices were assessed with the Parenting strategies for Eating and Activity Scale (PEAS) [29]. The PEAS consists out of five subscales: limit setting (6 items), controlling (6 items), monitoring (7 items), discipline (5 items) and reinforcement (2 items). | Never, almost never, sometimes, often, always. |
| Parenting Style | 14 (only the “warmth” scale)  36 | Parenting styles were assessed using the parental warmth scale and the controlling parenting style scale [28]. The parental warmth scale consists of six items. The controlling parenting style scale consists of five items. | Never, almost never, sometimes, often, very often |
