# Supplementary material for: Parenting support to prevent overweight during regular well-child visits in 0-3 year old children (BBOFT+ program), a cluster randomized trial on the effectiveness on child BMI and health behaviors and parenting
Source: PLoS One. 2020 Aug 18;15(8):e0237564. doi: 10.1371/journal.pone.0237564 (PMC7437453; doi:10.1371/journal.pone.0237564)
Supplement: S2 Table — (DOCX) [file pone.0237564.s003.docx]

**S2 Table. Analyses of non-response (n=1995).**

|  | Study non-completers  n=904 | Study completers  n=1091 | *p*-value^1^ |
| --- | --- | --- | --- |
| **Child characteristics** |  |  |  |
| Male gender (vs. female) (%) (missing n=37) | 456 (51.4%) | 564 (52.7%) | .555 |
| Birth weight in kilograms (mean, SD) (missing n=11) | 3442.5 (550.7) | 3471.4 (534.8) | .238 |
| Child is living with both parents (vs. single parent) (%) (missing n=26) | 864 (96.9%) | 1063 (98.7%) | **.007** |
| Ethnic background (%) (missing n=4)^2^ |  |  | **.001** |
| *Dutch* | 712 (79.1%) | 924 (84.7%) |  |
| *Non-Dutch* | 188 (21.1%) | 167 (13.9%) |  |
| **Characteristics of the mother** |  |  |  |
| Pregnancy duration in days (mean, SD) (missing n=76) | 276.9 (11.5) | 276.9 (11.4) | .983 |
| Age in years at child’s birth (mean, SD) (missing n=34) | 30.5 (4.6) | 31.3 (4.1) | **<.001** |
| BMI kg/m2 (mean, SD) (missing n=154) | 25.4 (4.5) | 25.2 (4.3) | .854 |
| Ethnic background (%) (missing n=4) |  |  | **.002** |
| *Dutch* | 774 (86.0%) | 988 (90.6%) |  |
| *Non-Dutch* | 126 (14.0%) | 103 (9.4%) |  |
| Education level (%) (missing n=39) |  |  | **<.001** |
| *Low* | 189 (21.6%) | 94 (8.7%) |  |
| *Mid* | 307 (35.0%) | 391 (36.2%) |  |
| *High* | 380 (43.4%) | 595 (55.1%) |  |
| **Characteristics of the father** |  |  |  |
| Age in years at child’s birth (mean, SD) (missing n=89) | 33.3 (5.5) | 33.7 (4.6) | .079 |
| BMI kg/m2 (mean, SD) (missing n=224) | 25.4 (3.6) | 25.2 (3.1) | .292 |
| Ethnic background (%) (missing n=25) |  |  | **.006** |
| *Dutch* | 758 (86.1%) | 983 (90.2%) |  |
| *Non-Dutch* | 122 (13.9%) | 107 (9.8%) |  |
| Education level (%) (missing n=111) |  |  | **<.001** |
| *Low* | 205 (24.3%) | 141 (13.6%) |  |
| *Mid* | 314 (37.2%) | 386 (37.1%) |  |
| *High* | 325 (38.5%) | 513 (49.3%) |  |
| **Study Condition** |  |  | .148 |
| *CAU* | 512 | 582 |  |
| *BBOFT+* | 392 | 509 |  |

^1^P-value based on independent t-test for continuous variables and Chi-square test for categorical variables to analyze the difference between the control and intervention group. ^2^ Ethnic background of the child was based on the grandparents’ country of birth.

Bold numbers indicate *p <.05.*
