# Supplementary material for: Parenting support to prevent overweight during regular well-child visits in 0-3 year old children (BBOFT+ program), a cluster randomized trial on the effectiveness on child BMI and health behaviors and parenting
Source: PLoS One. 2020 Aug 18;15(8):e0237564. doi: 10.1371/journal.pone.0237564 (PMC7437453; doi:10.1371/journal.pone.0237564)
Supplement: S1 Data — (PDF) [file pone.0237564.s004.pdf]

Primary prevention of overweight in preschool children

RESEARCH PROTOCOL

(July 1, 2008)

Dr. H. Raat

Dr. M. L'Hoir

Dr. M. Boere-Boonekamp

Dr. S. te Velde

Drs. M. Beltman

Drs. M. Struijk

PROTOCOL TITLE:

Primary prevention of overweight in preschool children

Protocol ID	120610018
Short title	Primary prevention of overweight
Version	2 (adapted on request of ZonMw)
Date	July 1, 2008
Coordinating investigator/project leader	*Dr. Hein Raat*<br>*Erasmus MC*<br>*Department of Public Health*<br>*P.O Box 2040*<br>*3000 CA ROTTERDAM*
Principal investigator(s) (in Dutch: hoofdonderzoeker/uitvoerder)<br>*Multicenter research: per site*	*Dr. Hein Raat (Erasmus MC)*<br>*Dr. M. L'Hoir (TNO Kwaliteit van Leven, Leiden))*<br>*Dr. M. Boere-Boonekamp (University Twente)*<br>*Dr. S. te Velde (VUmc)*
Sponsor (in Dutch: verrichter/opdrachtgever)	*ZonMW Prevention program*
Independent physician(s)	*Agnes van der Heide*<br>*Erasmus MC*<br>*Department of Public Health*

PROTOCOL SIGNATURE SHEET

Name	Signature	Date
Head of Department:<br>*Prof. dr. J. Mackenbach*		
Coordinating Investigator/Project leader/Principal Investigator:<br>*Dr. H. Raat*		

TABLE OF CONTENTS

Summary ... 5

1. Introduction and rationale ... 7
2. Objectives ... 9
3. Study Design ... 10
4. Study Population ... 16
5. Description of Interventions ... 18
6. Methods ... 27
7. Statistical Analysis ... 33
8. Ethical Considerations ... 34
9. Administrative aspects and publication ... 35

References ... 36

SUMMARY

This study aims to evaluate the effects on the prevention of overweight of three approaches that are currently being applied in Dutch preventive Youth Health Care (YHC) to parents of infants and toddlers: (a) ‘Usual care’, i.e. common advices regarding nutrition and playing of the infant and toddler; (b) 'BOFT+’, i.e. a training of YHC-nurses regarding their skills to advice parents regarding parenting; and (c) the ‘Healthy toddler', i.e. a specific advice to parents of toddlers at age 18 months and 24 months regarding nutritional habits, playing habits and watching TV, supported by a written advice generated by Internet.

Forty-five Youth Health Care (YHC) teams of circa10 regional YHC providers will participate in this study with net 1.500 children. The 45 teams will be assigned at random to (a) continue ‘Usual care’ (15 teams), or (b) to participate in the training for nurses regarding parenting skills (i.e. 15 ‘BOFT+’ teams), or to (c) YHC-teams that will apply the ‘Healthy toddler’ face-to-face consultation at age 18-months regarding nutritional habits, playing habits and watching TV, supported by an Internet generated, tailored written advice (15 teams). Informed consent to participate in this study will be obtained from the parents during a regular home visit by member of the YHC-team circa 2 weeks after birth; follow-up of the parents and their children is till the child is 36 months old.

According to the current YHC Overweight prevention protocol (‘Overbruggingsplan’) YHC-teams, in order to contribute to the prevention of overweight in childhood should promote 1) breastfeeding, 2) outdoor exercise, activity and play, 3) having breakfast daily, 4) few soft drinks and 5) less TV time (in Dutch: ‘B-BOFT’-behaviors).

(a)
‘Care as usual’ consists of common advices regarding nutrition and playing of the infant and toddler, and the above-mentioned ‘B-BOFT’ behaviors are part of such advices that are given according to the Product Description YHC, i.e. the regular scheme of YHC visits, e.g. through oral information and generic information leaflets.

(b)
The ‘BOFT+’ intervention is also based on this approach. However, the YHC-nurses in the ‘BOFT+’ teams will be trained how to provide parents with parenting skills necessary to act according to the recommended behaviors (e.g. only few TV time). In ‘BOFT+’, the YHC-nurses will have more knowledge and skills to augment usual care with explicit guidelines on childrearing. These guidelines are simple and encompass principles of stimulus control and modeling. YHC-workers will therefore be able to educate parents in how to set concrete boundaries for their children and how to create preconditions to make the child behave positively. The YHC-nurses will know how to advice parents regarding structuring time and space, how to implement rules and habits, how to be unambiguous and consistent and how to set clear boundaries. The nurses will be trained how to help parents controlling stimuli, and how unwanted behavior can be avoided. Parenting skills based on operant conditioning

will be incorporated in the training of professionals. Enlarging parenting skills is thought to be a precondition in order to change child-rearing styles.

(c)
The ‘Healthy toddler’ intervention is also based on the YHC-Overweight prevention protocol and has a focus on the promotion of parenting competence by face-to-face counseling, using a counseling style called ‘motivational interviewing’, strengthened by a dedicated E-health module. The 4 recommended behaviors, daily exercise/outdoor playing, family breakfast daily, few sugar sweetened drinks, minimal TV time (‘BOFT’), and associated parenting skills/attitudes will be assessed by the E-health module, prior to face-to-face counseling and result in a tailored advice, subsequently discussed during face-to-face counseling by a trained YHC-professional using motivational interviewing techniques. The E-health module includes a written reminder of useful information at approximately 1 month after the YHC-visit. The ‘Healthy toddler’ intervention will be provided twice to the parents/toddlers: at the age of 18 months and 24 months.

The study questions are:

1. Effect evaluation
What are the effects of (1) the ‘BOFT+’ intervention, applied from birth onwards and (2) the ‘Healthy toddler' intervention, applied when the child becomes a toddler, in terms of (a) improvement of energy balance-related behaviors, i.e. daily outdoor playing, daily family breakfast, few sugar sweetened drinks per day, and limited TV time per day, (b) in terms of optimal parenting practices/style, and (c) in terms of measures of body fatness at the population level, at follow-up at age circa 36 months?

2. Process evaluation
What is the adherence of parents and YHC professionals to the distinct elements of the ‘BOFT+’ intervention and the ‘Healthy toddler' intervention, how do they appreciate these elements, how often and to whom are these elements applied?

3. Cost-effectiveness evaluation
What is the ratio between costs and effects in terms of improvement of the four behaviors and potential future reduction in the prevalence of overweight of the ‘BOFT+’ and the ‘Healthy toddler' intervention compared to a control group receiving usual care?

We will explore differences in effects and process characteristics for subgroups of socially disadvantaged and non-Dutch children.

1. INTRODUCTION AND RATIONALE

In the Netherlands, preventive Youth Health Care (YHC - Jeugdgezondheidszorg) is committed to counsel parents regarding parenting competence, and to promote healthy development and growth for all children (Basistakenpakket Jeugdgezondheidszorg, 2002). These YHC-tasks are of utmost importance nowadays, as the prevalence of overweight and obesity among children has at least doubled in the past 25 years, especially in socially disadvantaged and specific ethnic subgroups (Fredriks 2005; Whitlock 2005).

Distinct elements of the Product Description YHC (Basistakenpakket JGZ) are being elaborated into national YHC consensus statements and eventually into evidence-based protocols (Standaarden Jeugdgezondheidszorg). Currently, consensus regarding overweight prevention has been reached through the YHC-Overweight detection protocol (Signaleringsprotocol overgewicht; Bulk-Bunschoten 2005) and the YHC-Overweight prevention protocol (Overbruggingsplan overgewicht; Hirasing 2005).

The YHC-Overweight prevention protocol consists of a strategy for primary prevention of overweight (i.e. promotion of healthy energy balance-related behaviors in the whole child population) and a strategy for secondary prevention of overweight (i.e. detection of children with established overweight and intensive counseling of these children and their parents).

In this protocol the effectiveness of two specific interventions regarding primary prevention of overweight and obesity will be evaluated in a clustered randomized trial. The two new interventions, ‘BOFT+’ and the ‘Healthy toddler’, are fully based on the YHC Overweight-prevention-protocol with a focus on promotion of parenting competence. They are easily applied in the YHC setting, but differ in content and complexity.

The control condition in the study is ‘care as usual’, i.e. care according to the Product Description YHC, i.e. the regular scheme of YHC visits during which general information regarding nutrition, playing and exercise is provided, e.g. through oral information and generic information leaflets, acknowledging the knowledge of the YHC-Overweight Prevention Protocol that states that breastfeeding is preferred over bottle feeding and that parents should promote healthy nutrition and exercise (Basistakenpakket Jeugdgezondheidszorg, 2002; Hirasing et al., 2005).

In intervention arm (a) of the study, the BOFT+ intervention aims at implementing healthy life-style habits continuously, from early age on (from birth on) to three years of age. The nurses of the YHC-teams that participate in the BOFT+ intervention will be trained in how parents can be supported in general parenting skills. This training is based on results of a previous study on determinants of behavior that is related to overweight in young children (Boere-Boonekamp, 2005; ZonMw Grant # 50-50140-98). The approach focuses on training the YHC-nurses how to help parents with the transition to a new phase in life, namely that of a young parent; people are more receptive to changes during such transitional moments. In

this arm the education and counseling of parents will not only focus on eating habits and exercise, but also on childrearing issues, by using elements of the learning theory and enlarging parental sensitivity together with high expectations concerning the possibility of self-control of the child (the so called authoritative parenting style). YHC nurses will apply the new approach during each YHC-contact with parent(s) in the preschool period. Examples of the techniques that will be used are: modeling, stimulus control, and intermittent confirmation.

In intervention arm (b) of the study, the ‘Healthy toddler’ intervention will be provided by YHC professionals to parents 2 times: (1) at the age of approximately 18 months, and (2) at the age of approximately 24 months. The nurses that participate in the ‘Healthy toddler’ intervention will apply motivational interviewing techniques during the face-to-face consultation and address child-rearing issues related to healthy habits (Hirasing et al., 2005). The focus is on 4 priority behaviors (‘BOFT’): (1) Promotion of daily exercise and outdoor playing time; (2) Having a family breakfast daily; (3) Lessening intake of sugar sweetened drinks; (4) Limitation of TV time (in Dutch 'BOFT': Buiten spelen, Ontbijten, Frisdrankgebruik ontmoedigen, TV kijken). These behaviors and associated attitudes are assessed by an E-health module resulting in a tailored advice, subsequently discussed during face-to-face counseling. The E-health module includes a reminder circa 1 month after face-to-face counseling. The module was developed and implemented in ZonMw project # 4010.0033, named 'The start of healthy living'. The combination of prior on-line assessment, tailored advice, face-to-face counseling, and an E-health ‘booster’ is a promising combination (Mangunkusumo, 2006).

Context analysis and related studies

Given the epidemic increase of relative overweight and obesity in childhood (Whitlock 2005), all involved parties are fully aware of the necessity of effective prevention strategies, are therefore willing to contribute to the study, and eager to learn and implement the results.

January 2007, parties that collaborate in the current study, started a ZonMw cluster-randomized trial to evaluate the YHC-Overweight detection protocol (Bulk-Bunschoten 2005) in combination with the YHC-Overweight prevention protocol (Hirasing 2005), at the child age of 5 years, i.e. school-going children, with a focus on selection of children with overweight, i.e. secondary prevention.

From the field of YHC several requests emerged to evaluate, in addition, the Overweight-prevention protocol with the focus on primary (not secondary) prevention of overweight by YHC-nurses and physicians among preschool children. The argument is that primary prevention is necessary, and probably effective, in very young children who are in the process of developing their habitual energy balance-related behaviors; once these behaviors have been established it is considered to take more effort to induce change (Hirasing 2005).

In the past months, parties of the current study have developed an educational course for YHC-nurses and -physicians based on the previous study on determinants of behavior related to overweight in young children (Boere-Boonekamp 2005; ZonMw Grant # 50-50140-98 and Fonds OGZ). A pilot of the first part of this course is planned for October 10, 2007. After adaptation, this part of the course can be used in the ‘BOFT+’ research arm of the project at hand. Recently, an application for development of a comparable course for teachers of preschool and nursery facilities was submitted to ZonMw as well (continuation of the Fonds OGZ project).

The primary prevention element of the YHC-Overweight prevention protocol, to be evaluated in this study, has broad support among policy makers and managers as well as among the YHC professionals, including the YHC-nurses.

2. OBJECTIVES

In this study we will evaluate the effects of 2 distinct primary prevention strategies regarding overweight in preventive Youth Health Care (YHC) for preschool children (Jeugdgezondheidszorg 0-4 jarigen): (a) the ‘BOFT+’ intervention with - as primary starting point - a focus on child rearing issues that are relevant for the development of behaviors related to overweight, starting at birth and using elements of learning theory, stimulus control and modeling (based on ZonMw project # 50-50140-98); (b) The ‘Healthy toddler' intervention with - as primary starting point - a focus on behaviors related to overweight using E-health in combination with face-to-face counseling and motivational interviewing for parents of young toddlers (18-24 months) (based on ZonMw project # 4010.0033). Both are based on the broadly accepted YHC-Overweight-prevention-protocol, saying that five behaviors should be promoted: breast feeding, daily exercise/outdoor playing, family breakfast daily, few sugar sweetened drinks, minimal TV time (so-called ‘B-BOFT’ behaviors). The control condition in the study implies ‘usual care’ according to the Product Description YHC with general information regarding nutrition, playing and exercise, e.g. through oral information and generic information leaflets.

Study questions:

Effect evaluation

1. What are the effects of (a) the ‘BOFT+’ intervention applied from birth onwards and (b) the ‘Healthy toddler' intervention applied when the child becomes a toddler, in terms of improvement of energy balance-related behaviors, i.e. daily outdoor playing, daily family breakfast, few sugar sweetened drinks per day, and limited TV time per day (in Dutch 'BOFT' behaviors), in terms of optimal parenting practices/style, and in terms of measures of body fatness at the population level, at follow-up at age circa 36 months?

Process evaluation

2. What is the adherence of parents and YHC professionals to the distinct elements of the 'BOFT+' intervention and the 'Healthy toddler' intervention, how do they appreciate these elements, how often and to whom are these elements applied?

Cost-effectiveness evaluation

3. What is the ratio between costs and effects in terms of improvement of the four behaviors and potential future reduction in the prevalence of overweight of the 'BOFT+'- and the 'Healthy toddler' interventions compared to a control group receiving usual care?

We will explore differences in effects and process characteristics (e.g. adherence) for subgroups socially disadvantaged and non-Dutch children.

3. STUDY DESIGN

In this section we address the design of the study, the details of (a) the 'BOFT+' intervention and (b) The 'Healthy toddler' intervention (i.e. the theoretical framework and content), the outcome measures of the study, the procedure of the study (including inclusion of participating centers, randomization procedure, training of the participating Youth Health Care-teams (YHC-teams), inclusion of parents/families in the study), power considerations regarding the differences in outcomes between each Intervention group and the Control group that can be detected in this study, the proposed statistical analyses of the results, and the time schedule for the study.

In this study we will evaluate (a) the 'BOFT+' intervention with - as primary starting point - a focus on child rearing issues that are relevant for the development of behaviors related to overweight, starting at birth, increasing self esteem of parents, anticipating on parenting by reinforcing all positive parenting strategies from early age on, using the relatively simple elements of motivational interviewing (empathy and no use of warnings, confrontations, discussion), using elements of mediation therapy and using elements of learning theory, stimulus control, modeling and operant conditioning (based on ZonMw project # 50-50140-98); and (b) The 'Healthy toddler' intervention with - as primary starting point - a focus on behaviors related to overweight using E-health in combination with face-to-face counseling and motivational interviewing for parents of young toddlers (18-24 months) (based on ZonMw project # 4010.0033). The 'Healthy toddler E-health module provides tailored advice to parents regarding healthy behaviors of their toddler and related child rearing practices; the face-to-face counseling addresses issues raised by the E-health module, including questions that the parents may have, using motivational interviewing techniques as proposed by the YHC-Overweight-prevention-protocol (Hirasing et al, 2005).

(a) ‘BOFT+’ intervention

The ‘BOFT+’ intervention has - as primary starting point - a focus on child rearing issues that are relevant for the development of behaviors related to overweight. By doing so it aims at implementing healthy life-style habits continuously, from early age on (from birth on) during guidance offered to parents by YHC in the whole preschool period. An authorative parenting style means high levels of effective parenting, not many inter-parental conflicts en high parental self-efficacy. A healthy lifestyle can be reached by healthy eating, physical activity and the use of positive parenting. In the current study the effect of the intervention up to (and including) age 24 months old will be evaluated with a final follow-up measure at age 36 months.

The strategy of the ‘BOFT+’ intervention is based on results of a previous study on determinants of behavior that is related to overweight in young children (Boere-Boonekamp 2005; ZonMw Grant # 50-50140-98). In this intervention the education and counseling will not only focus on eating habits and exercise, but also on childrearing issues, by using elements of the learning theory and enlarging parental sensitivity together with high expectations concerning the possibility of self-control of the child (the so called authoritative parenting style. YHC nurses will apply the new approach during each YHC-contact with parent(s) in the preschool period. Examples of the techniques that will be used are: modeling, stimulus control, operant conditioning such as intermittent confirmation.

(b) The ‘Healthy toddler’ intervention

The ‘Healthy toddler’ intervention has - as primary starting point - a direct focus on four behaviors related to overweight as defined in the YHC-Overweight-prevention-protocol that start to be of relevance in young toddlers: daily exercise/outdoor playing, family breakfast daily, few sugar sweetened drinks, minimal TV time (so-called ‘BOFT’ behaviors). Child rearing issues related to these behaviors are addressed as well in the ‘Healthy toddler’ intervention.

The ‘Healthy toddler’ intervention consists of an E-health module with tailored advice to parents regarding the ‘BOFT’ behaviors of their toddler and related child rearing practices, which is combined with face-to-face counseling in the setting of regular YHC at the child age of circa 18 months for the first time and at child age of circa 24 months for the second time. The E-health module is offered prior to both visits, and is repeated, as a reminder, a month after both visits. The face-to-face counseling will address specific issues raised by the E-health module, including questions that the parents may have. As proposed by the YHC-Overweight-prevention-protocol YHC-professionals will make use of motivational interviewing techniques in the two counseling sessions. As specific YHC-visiting schemes vary by region in the Netherlands, some variation will be allowed regarding age of application.

The study is designed as a cluster-randomized trial (c-RCT) with two intervention conditions (see above) and a control condition (‘usual care’), with inclusion shortly after child birth, repeated measurements prior to and during the interventions, and a follow-up measurement at age 36 months, which is circa 12 months after the last intervention contact with the parents around the age of 24 months old in both intervention groups. The YHC-team (Consultatiebureau-team) is the unit of randomization in order to avoid contamination between the intervention condition and the control group condition within teams (Campbell et al., 2004).

Circa ten regional services providing Youth Health Care for preschool children (in Dutch: Jeugdgezondheidszorg 0-4 jaar, or Ouder- en Kindzorg) from metropolitan and non-metropolitan areas, to be selected in an open procedure, will participate in the study with a total of 45 Youth Health Care teams (Consultatiebureau-teams). Within each service, YHC-teams will randomly be assigned to either one of the two intervention groups or the control group. The last follow-up measurement in the study that we propose now will take place at the age of circa 36 months, during a regular visit to the YHC center in combination with a self-report questionnaire. A process and cost-effectiveness evaluation will accompany the effect evaluation. When the study is successful, follow-up projects with continuation of the interventions and/or follow-up measurements at older age will be proposed at ZonMw or other funding organizations in the future.

Figure 1. ***Flowchart of the study***

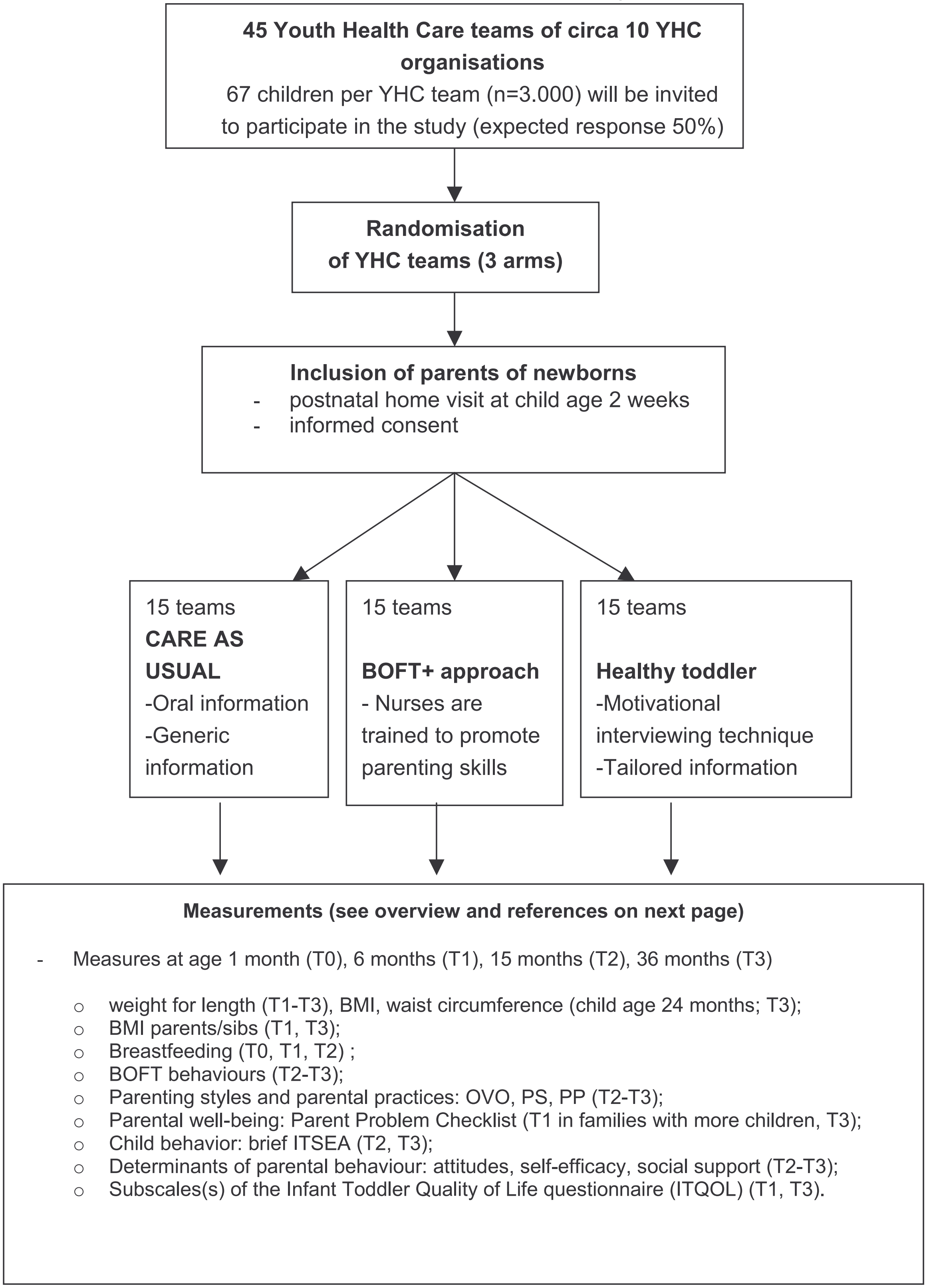

Overview and references regarding the measurement instruments in the study

International BMI cut- off points

- Cole et al. Establishing a standard definition for child overweight and obesity worldwide: international survey. BMJ. 2000 May 6;320(7244):1240-3.

Parent Problem Checklist (PPC)

- Dadds MR, Powell MB. The relationship of interparental conflict and global marital adjustment to aggression, anxiety and immaturity in aggressive and nonclinic children. Journal of abnormal child psychology, 1991; 19:553-567.
- Arnold EH, O'Leary SG, Wolff LS, Acker MM. The parenting scale: a measure of dysfunctional parenting in discipline situations. Psychological assessment 1993;5:137-144.

Opvoedingsvragenlijst Voor Ouders (OVO) (child rearing questionnaire for parents)

- Block. The child rearing practices report: A technique for evaluating parental socialization orientations. Berkeley, CA: University of California Institute of Human Development, 1965.
- Dekovic, M., Janssens, M.A.M., & Gerris, J.R.M. (1991). Factor structure and construct validity of the Block Child Rearing Practices Report (CRR). Psychological Assessment, 3,182-187.

Parenting style (PS)

- Arnold EH, O'Leary SG, Wolff LS, Acker MM. The parenting scale: a measure of dysfunctional parenting in discipline situations. Psychological assessment 1993;5:137-144.

Parenting practices and rules (PP)

- Arredondo et al. Is parenting style related to children's healthy eating and physical activity in Latino families? Health Educ Res. 2006 Dec;21(6):862-71,

Energy balance-related behaviors (so-called 'BOFT' behaviors) and the determinants of these behaviors in families

- In a recent ZonMw trial regarding secondary prevention of overweight in young children we developed a parent questionnaire to measure the prevalence of energy balance-related behaviors (so-called 'BOFT' behaviors) of young children and the determinants of parental behaviors regarding 'BOFT', based on the Theory of Planned Behavior: H. Raat et al., Vragenlijst voor ouders Leefstijl, groei en gezondheid van kinderen. Erasmus MC, Rotterdam, 2007. Hierbij is gebruik gemaakt van:
- Renders CM, et al, Televisiekijken en enkele eetgewoonten bij Amsterdamse 6-14-jarigen; een transversaal onderzoek. Ned Tijdschr Geneeskd 2004, 148(42):2072-2076.
- O'Connor TM, Yang S-J & Nicklas TA. Beverage Intake Among Preschool Children and Its Effect on Weight Status. Pediatrics 2006; 118, e1010-1018.
- Conner M and Sparks P, Theory of planned behaviour and health behaviour, in: Predicting Health Behaviour, Conner M and Norman P, Open University Press, Berkshire, UK, 2005.

- Vries, H. de, Dijkstra, M., Kuhlman, P. Self-efficacy: the third factor besides attitude and subjective norm as a predictor of behavioural intentions. Health education research 1988; nr.3, pp. 273-282.
- Sutton S. Stage theories of Health Behaviour. In: Predicting Health Behaviour, Conner M and Norman P, Open University Press, Berkshire, UK, 2005.
- Laar CWF van de, Renders CM, Hirasing RA: Preventie van overgewicht: een minimale interventie strategie bij 5/6 jarige kinderen binnen de JGZ. NWO-werkgemeenschap Jeugd en Gezondheid: 2006.

Health and well-being parent (SF-12)

- Gandek, B., J. E. Ware, et al. (1998). Cross-validation of item selection and scoring for the SF-12 Health Survey in nine countries: results from the IQOLA Project. International Quality of Life Assessment. J Clin Epidemiol 51(11): 1171-8.

Health and well-being child (ITQOL, and brief-ITSEA)

- Raat, H., J. M. Landgraf, et al. (2007). Reliability and validity of the Infant and Toddler Quality of Life Questionnaire (ITQOL) in a general population and respiratory disease sample. Qual Life Res 16(3): 445-60.
- The brief Infant-Toddler Social and Emotional Assessment (brief ITSEA; 42 items) suitable for children 12 to 36 months of age, Briggs-Gowan MJ et al, The Brief Infant-Toddler Social and Emotional Assessment: screening for social-emotional problems and delays in competence. J Pediatr Psychol, 2004. 29(2): p. 143-55.

TIME SCHEDULE IN MONTHS

The schedule below is provided in months from the start of the project, which is scheduled at July 1, 2008.

0-6: Recruiting 45 YHC-centers, preparing procedures/measures/protocols, and (start of) training of YHC-professionals regarding ‘BOFT+’- (15 YHC-teams) and the ‘Healthy toddler’ (15 YHC-teams) interventions.

6-18: During a year 3.000 parents of newborns will be provided information about the study and will be asked informed consent by 60 YHC-teams (expected participation rate at least 50%).

6-19: After recruitment of 1.500 parents/newborns, baseline measurements (T0 at child age 1 month) will performed in the 3 study arms.

6-19: In the ‘BOFT+’ study arm (15 YHC-teams) the ‘BOFT+’ intervention will be started regarding 500 parents/newborns at child age 1 month.

12-24: Measurements at child age 6 months in 1.500 parents/infants (T1).

21-33: Measurements at child age of 15 months in 1.500 parents/infants (T2).

23-37: In the ‘Healthy toddler’ study arm (15 YHC-teams) the first part of the ‘Healthy toddler’ intervention will be applied to 500 parents/toddlers at child age of circa 18 months.

29-43: In the ‘Healthy toddler’ study arm (15 YHC-teams) the second part of the ‘Healthy toddler’ intervention will be applied to 500 parents/toddlers at child age of circa 24 months.

30-42: In the ‘BOFT+’ study arm (15 YHC-teams) the last scheduled contact moment between YHC-team and parents regarding the ‘BOFT+’ interventions will take place regarding 500 parents/toddlers at child age 24 months.

42-54: Measurements at child age of 36 months in 1.500 parents/infants (T3).

6-55: Data entry, and data cleaning.

18-60: Analyses, dissemination of the results.

4. STUDY POPULATION

The research team will recruit circa ten services providing Youth Health Care for preschool children, with a total of 45 YHC-teams (Consultatiebureau teams) that participate in the study. The participating research centers (Erasmus MC in Rotterdam, TNO in Leiden, TU Twente in Enschede and VUmc in Amsterdam) have excellent relations with the Youth Health Care providers in the Netherlands, within and without the context of the recently established ‘Academic Workplaces for Public Health’ (Academische Werkplaatsen Publieke Gezondheidszorg), and foresee no difficulties in recruiting a total of 45 YHC-teams that will participate in the study.

The study population includes parents and their babies/toddlers, eligible for the regular YHC visits from birth until elementary school age (preschool YHC; Consultatiebureau zorg), during the study, that belong to one of the participating YHC services. Parents included in the study must be able to speak Dutch; severely handicapped toddlers are excluded from participation. Representative groups of parents, including those with relatively low education and from ethnic minority groups, will be included in the study. Parents will be fully informed about the study and informed consent will be obtained prior to inclusion in the study. Parents will be informed about the study when the YHC nurse visits the young parents in the second week after birth; informed consent may be provided during the first visit at the YHC center four weeks after birth. Approval of the Medical-Ethical Committee of Erasmus MC – University Medical Center Rotterdam will be obtained.

Intervention group (a)

The intervention group consists of parents of preschool children that participate in the study and receive preventive Youth Health Care from one of the 15 YHC-teams that is trained to administer the 'BOFT+' intervention that is described below. The 'BOFT+' intervention consists of several elements (see below), but is administered - in principle - at all regular YHC-visits.

Intervention group (b)

The intervention group consists of parents of preschool children that participate in the study and receive preventive Youth Health Care from one of the 15 YHC-teams that is trained to administer the 'Healthy toddler' intervention that is described below. The 'Healthy toddler' intervention is applied twice, at child-age of circa 18 months and circa 24 months old.

Control group

The control group consists of parents of preschool children that participate in the study and receive preventive Youth Health Care from one of the 15 YHC-teams that apply 'usual care'. 'Usual care', the control condition in this study, implies care according to the Product Description YHC, i.e. the regular scheme of YHC visits during which general information regarding nutrition, playing and exercise is provided, e.g. through oral information and generic information leaflets, acknowledging the knowledge of the YHC-Overweight Prevention Protocol that states that parents should apply the so-called 'B-BOFT' behaviors to prevent the development of overweight, i.e. breast feeding, daily exercise/outdoor playing, family breakfast daily, few sugar sweetened drinks, minimal TV time (Basistakenpakket Jeugdgezondheidszorg, 2002; Hirasing et al., 2005).

POWER CONSIDERATIONS AND DETECTABLE DIFFERENCES

We expect to receive complete data of 1.050 parents/toddlers at follow up at age 36 months old, equally divided over the 2 intervention groups and the control group. We assume equal standard deviations in the intervention groups and the control group, alpha of 0.05 and power of 0.80. We applied a correction factor to account for the cluster design, assuming an average cluster size of 23,3 toddlers (1.050/45) and an intra-class correlation coefficient of 0.10. For this expected sample size and assumptions, we calculated minimal detectable differences between an intervention and the control group at follow-up. We applied standard deviations and proportions/percentages reported in the literature or currently available data from the prior ZonMw projects (# 50-50140-98; # 4010.0033).

Target behaviors at age 36 months old:
Regarding the mean number of the reported minutes of physical activities of the children per day at age 36 months old, the study can detect a difference between intervention/control group of 18 minutes/day (SD=52 min/day). Regarding the mean consumption of sugar-sweetened drinks in glasses per day, a difference of 0.5 glasses/day (SD=1.5 glasses/day) can be detected. Regarding the mean number of minutes of TV viewing per day, a difference of 11 minutes/day can be detected (SD=32 minutes/day).

BMI at age 36 months old:
A difference of 0.44 BMI points between the mean BMI in the intervention group and the mean BMI in the control group at age 36 months can be detected by the study (SD=1.3) (Atkin & Davies, 2000).

Since we will use repeated measures (at age 15 months respectively 24 months in addition to 36 months) as covariates in the analyses, the power will be higher than indicated, which will facilitate the exploration of interaction effects of child gender, social disadvantage, ethnic background, and parental overweight status.

5. DESCRIPTION OF THE INTERVENTIONS

In addition to the descriptions above, in this paragraph we describe the theoretical framework of (a) the ‘BOFT+’- and (b) The ‘Healthy toddler’ interventions that will be evaluated in this study. The content of both interventions will be described in the text, as well as in an appendix with a schematic overview of both ‘BOFT+’- and the ‘Healthy toddler’ intervention.

A. THE ‘BOFT+’ INTERVENTION

The ‘BOFT+’ intervention is intended to promote the key behaviors for the prevention of overweight in children as defined in the YHC-Overweight Prevention Protocol from early age on: 1. Promotion of breastfeeding, 2. Promotion of outside exercise, activity and play, 3. Promotion of having breakfast, 4. Discouragement of soft drinks, and 5. Discouragement of television viewing. Promotion of breastfeeding has a specific context, is mainly limited to the first year of life, and is not the main focus of the ‘BOFT+’ intervention. Behaviors 2-5 relate to child rearing issues that are the primary focus of ‘BOFT+’; these child-rearing issues are introduced from birth on in the ‘BOFT+’ intervention.

The ‘BOFT+’ intervention includes targeted education that anticipates on common problems and explains the basics of stimulus control and classic conditioning in order to enable parents to create the conditions that stimulate desired behavior in the child. Concrete examples and advice on the subject of exercise and eating habits, derived from our previous study that may prevent weight problems in young families will be incorporated (Boere-Boonekamp 2005).

Theoretical framework of the 'BOFT+' intervention

The 'BOFT+' intervention focuses on parenting styles used within the families. It is essential that health care workers in the YHC centers (well-baby clinics) are educated about social learning principles, the different parenting styles and the associated risks. Furthermore, they have to be able to disseminate this knowledge, know how to coach parents in achieving competent parenting. Social learning principles can be dichotomized into classic and operant conditioning.

Specific behavior is caused by antecedent factors. One tries to intervene before the unwanted behavior occurs, and to create conditions suitable for positive behavior. Parents learn that it is important to set clear boundaries, have unambiguous rules. They receive suggestions to help structure time and space, how to introduce ground rules, and how to state these rules clearly, consistently. Parents should collaborate as a team in defining the rules and applying them consistently. By controlling the stimuli in this manner, disruptive behavior can be avoided (Bosch 1997). These principles are the basis of the learning theory.

Three targets of the BOFT+ approach are:
a. Enlarge parenting skills;
b. Optimize nutrition;
c. Optimize physical activity.

Ad a. Enlarging parenting skills is operationalized as:

-increase children's self esteem by spending time with the child, talk and show affection to the child. Regularly interact with a child while it is behaving positive, and talk with children about their activities, cuddle, touch and hold the child (bodycontact).
-encourage healthy behaviors: reinforce positive behavior by praising the specific behavior
-give positive attention: offer non-verbal attention (smiling, touching)
-offer attractive activities: play material etc. (age and development phase related)
-parent is positive example (modeling);
-reinforce spontaneous learning moments
-reinforce learning by experience, instruction

Stimulus control
Basic rules and regulations (age en development phase related)
Direct intervention when child is misbehaving
Clear instructions in a quiet way

Operant conditioning
Misbehavior will be related to direct consequence
Time out

Ad b. Optimize nutrition is operationalized as:

-establishing eating routine (0-9 months regularity and stimulus reduction (Sleuwen van, 2006); > 6 months specific times for meals and snacks (stimulus control);
-prevention of introduction of extra foods (commercial snack foods, take aways, sugar drinks)
-provision of healthy alternatives

ad c. Optimize physical activity is operationalized as:

-prevention of sedentary activities (TV etc.)
-increase physical activity (daily walk, play-pen, play outside etc.)

These three intervention targets are related to the child's body size, food intake, child behavior and parenting. They are described in detail by age (and developmental phase) in the Annex and in the report: "Ingredients of an intervention" based on the determinant study conducted by Boere-Boonekamp e.a. (2005; ZonMw Grant: # 50-50140-98).

Parents will enlarge their parenting skills by increasing children's self-esteem, encouraging healthy behavior by setting a good example and using praise and reward in the correct way and by managing problem behaviors by setting ground rules, clear instructions and the use of consistent consequences (Bakker & Husmann 1994; West 2007).

The 'BOFT+' intervention is relatively simple and easily communicated by YHC workers to parents. In this intervention behavioral goals are set out: these goals will be partially derived from our earlier study on behavior in young families that puts children at risk of weight problems (Boere-Boonekamp 2005). The goals cover specific issues of eating behavior, exercise and activities. The intervention targets parenting skills in the context of everyday, naturally occurring examples. Specific anticipating parenting education will be given during the standard YHC (well-baby) visits. The principles of stimulus control, modeling and operant conditioning will be taught (ZonMw project # 50-50140-98).

Please refer to the scheme (below, Figure 2) of the specific elements of the 'BOFT+' intervention according to child age.

Figure 2. Flowchart of the ‘BOFT+’ intervention.

Activity by YHC professional	Theme	Age of the child
Targeted education anticipating on common problems and explaining the basics of stimulus control, classical conditioning, modelling, operant conditioning.	- Breastfeeding, bottle feeding (amount, extra powder, self-regulation);<br>- Taste development;<br>- Use of playpen and television.<br>- Crying and sleeping rhythm	± 3 months
	Reminder of the above themes.	± 4 months
	Reminder of the above themes.	± 6 months
Targeted education anticipating on common problems and explaining the basics of stimulus control, classical conditioning, modelling, operant conditioning.	- Introduction of sweetened drinks;<br>- Number of eating moments;<br>- Crying and sleeping rhythm<br>- Television viewing.	± 7,5 months
	Reminder of the above themes.	± 9 months
Targeted education anticipating on common problems and explaining the basics of stimulus control, classical conditioning, modelling, operant conditioning.	- How to eat with a child at the dinner table;<br>- Going outdoor for a walk, playing outdoors;<br>- Sleeping rhythm;<br>- Television viewing.	±11 months
Targeted education anticipating on common problems and explaining the basics of stimulus control, classical conditioning, modelling, operant conditioning.	- Television viewing	± 14 months

B. *THE 'HEALTHY TODDLER' INTERVENTION*

Theoretical framework of the 'Healthy toddler' intervention

While the 'BOFT+' intervention - as primary starting point - focuses on child rearing issues from birth on, the 'Healthy toddler' intervention - as primary starting point - focuses on the established key-behaviors related to child-overweight. These are:

1. Promotion of outside exercise, activity and play,
2. Promotion of having family breakfast daily,
3. Discouragement of sweet drinks, and
4. Discouragement of television viewing (Hirasing et al., 2005).

The approach applied is the social-ecological model (Green et al., 1996), which recognizes the importance of environmental and personal factors on health behavior.

This guided the development of the 'Healthy toddler' intervention in the recently finished ZonMw project # 4010.0033, named 'The start of healthy living'. In case of very young children, parents can be considered as the main mediators of the toddler's environment. Parents influence the social and physical environment through their general parenting style (Darling & Steinberg, 1993) and parental practices. Fostering an authoritative parenting style has shown to decrease food stimuli in home (Golan et al., 2006). Therefore, parental practices have to be addressed in order to change the toddler's behavior. Important determinants of parental behavior can be found in classical behavior change theories, such as the Theory of Planned Behavior (Ajzen & Madden, 1986) and Social Cognitive Models (Bandura, 1986), and include attitude, social support, self-efficacy, perceived barriers, knowledge, and awareness. Therefore, the aim of the 'Healthy toddler' intervention is to make parents aware of potential/future overweight related problems and be motivated to prevent the development of overweight in their children from early age on by improving the four key behaviors defined in the YHC-Overweight Prevention Protocol, through their parenting.

In the 'Healthy toddler' intervention, positive attitudes of the parents towards applying strict behavior and positive attitudes towards the specific, recommended, parental practices are being promoted. Furthermore, parents are supported to overcome barriers for not applying these practices, and parents learn specific skills in order to be able to change their parenting according to the recommendations of the YHC-Overweight Prevention Protocol (Hirasing et al., 2005).

According suggestions proposed by McGuire for successful communication, several steps have to be taken in order to achieve behavior change (McGuire, 1985). First, the topic at hand (i.e. prevention of potential/future overweight related problems from early age on) has to be brought to attention and made understood. Second, determinants of the behavior have to be changed, e.g. creating a positive attitude towards the recommended behavior, increase

self-efficacy to perform the behavior, in order to change behavior. Finally, behavior change has to be maintained using feedback and reinforcement. The ‘Healthy toddler’ intervention package follows this scheme by application of the different elements included in this intervention package (see below; also see Annex with overview of the ‘Healthy toddler’ intervention).

Briefly, the intervention package will be applied twice (at age circa 18 months and age circa 24 months) and consists of an E-health module and face-to-face counseling by the YHC-professional. At the first application, at child age of circa 18 months, the E-health module will be introduced to the parents along with the regular invitation for the scheduled YHC-visit. It brings the topic to attention of the parents prior to visiting the YHC-center in an understandable manner and makes parents aware of the potential/future problem. Results from the ‘Healthy toddler’ pilot study show that the majority of the parents indeed understands the message and thinks it is useful and applicable. During the subsequent face-to-face counseling at the YHC-center for which an extra 15-minutes has been allocated for this topic, the YHC professional increases knowledge, awareness, attitudes and self-efficacy with regard to strict parenting practices. The YHC professionals, specifically trained for this purpose (see below), will apply motivational interviewing, which has proven to be effective in changing health behaviors in various settings and has been adapted for this specific YHC application (Rollnick et al., 2005). Finally, a second element of the E-health module will be applied, i.e. a reminder circa one month after the YHC-visit to maintain the behavior change, using positive feedback and reinforcement.

At the second application of the ‘Healthy toddler’ intervention package at the child age of circa 24 months, this series of activities (E-health module prior to the YHC-visit, face-to-face-counseling at the YHC center, E-health reminder one month afterwards) will be repeated with a focus on behavior change maintenance, or, in the case of relapse, the same approach as applied at age of circa 18 months old. At age 24 months, 10-minutes extra time will be allocated for this specific topic during face-to-face counseling.

The distinct elements of the ‘Healthy toddler’ intervention
As specific YHC-visiting schemes vary by region in the Netherlands, some variation will be allowed regarding the age of the first (circa 18 months) and second (circa 24 months) application of the intervention package, with a minimum age of 14 months old.

** The E-health module 1 month prior to YHC-visit (at child age circa 18 months)

Prior to the scheduled YHC-visit at child age of circa 18 months old, along with the regular invitation for the YHC-visit, after having obtained informed consent, parents are invited to complete an E-health module. In 2007, circa 80% of Dutch households of 25-45 year olds have Internet access (CBS, 2007). Therefore, in the context of this study, we have decided that parents may decide themselves whether they gain access to the E-health module through Internet with immediate online tailored feed-back, or whether they use the E-health

module by mailed materials (after data-entry of the questionnaire upon receipt, the tailored feed-back will be mailed back to their homes directly).

The E-health module assesses the four priority behaviors defined by the YHC-Overweight Prevention Protocol, the general attitudes of the parents toward overweight prevention from early age on, and whether parents apply the recommended behavior specific parental practices. Participants will receive feedback tailored to their personnel situation given the provided answers posed by the E-health module (online or by mail if parents prefer so). As part of the tailored feedback, current behavior will be compared with the recommendations for each of the four priority behaviors. This will increase knowledge of the recommendations and make parents aware of their child’s behavior. Additionally, parents will receive tailored advice on how to change their child’s behavior, taking into account the recommended behavior specific parental practices (e.g. setting rules) (Hirasing et al, 2005).

The information entered by the parents regarding the four target behaviors and a summary of their attitudes generated by the E-health module will be sent to the YHC professional prior to the visit, enabling the professional to use this information in the counseling.

** Face-to-face counseling at the YHC-center (child age circa 18 months)

During the regular visit of parents and their (circa) 18 months old toddler to the YHC center, the YHC professional will apply the Overweight Prevention Protocol and make use of available information from the E-health module. The face-to-face consultation regarding overweight prevention will be performed by a trained YHC-professional of the intervention-team. At this first time face-to-face counseling at the YHC-center, 15 minutes extra time will be allocated for this topic in the context of the regular YHC visit (including measurements for the purpose of the evaluation) (see Korfage et al, 2002).

The Overweight Prevention Protocol consists firstly of a structured assessment of the four priority energy-balanced behaviors. In the face-to-face counseling, the trained YHC-professional will, according to the YHC-Overweight prevention protocol, apply motivational interviewing to promote daily exercise and outdoor playing time, having a family breakfast daily, lessening sugar sweetened drinks, and limiting TV time (in Dutch 'BOFT' behaviors) (Hirasing et al., 2005). See below regarding the training of the YHC-professionals in this Intervention group.

During the counseling, the YHC-professional will motivate parents to prevent the development of overweight in their child while it grows up. The YHC-professional will make parents see advantages of applying strict parental practices, and correct false assumptions parents have with regard to applying strict rules. Additionally, he or she will help identifying barriers experienced by the parents preventing them to perform the preferred parenting behavior and provide a different perspective on the barrier in order to overcome the obstacle.

Self-efficacy will be improved by providing them with skills to apply strict parental practices (Sanders et al., 2003; West, 2005).

Parenting skills that the YHC professional will include in the counseling interview are: establishing eating routines by setting specific times for meals and snacks, especially for having breakfast daily; reducing extra foods such as sugar sweetened beverages; provide healthy alternatives, like fruits and low fat/sugar foods; demand daily consumption of fruit and vegetables, limit sedentary activities by setting rules about TV viewing and increase physical activity by active family leisure time and active transport (Hirasing et al, 2005).

** E-health module reminder 1 month after YHC-visit

Circa one month after the face-to-face counseling at the YHC-center, parents will receive an electronic (or paper version if they prefer so) reminder regarding their tailored advice; including the tailored advise with regard to behavior specific parenting practices.

** Repetition of the 'Healthy toddler' intervention package at child age (circa) 24 months

The above-described distinct elements of the 'Healthy toddler' intervention package will be applied again at the age of (circa) 24 months. The E-health module, one month prior to the second counseling on this topic at the YHC-center, can assess if behavior change was achieved and provide positive feedback and reinforcement if this was the case. If not, parents will again receive tailored advice on how to achieve behavior changes. During the second-time face-to-face counseling regarding this topic, the YHC professional will assess if behavior change was maintained. If this was the case, the YHC professional will provide positive feedback and reinforcement. If behavior change was not achieved or parents reported relapse, the YHC professional will assess what causes the relapse and again apply the same counseling strategy, based on a motivational interviewing approach, as described above.

At this second time face-to-face counseling at the YHC-center, 10 minutes extra time will be allocated for this topic in the context of the regular YHC visit (including measurements for the purpose of the evaluation) (see Korfage et al, 2002).
The E-health reminder circa one month after the second face-to-face counseling, will also be used for either positive feedback, or provision of tailored advice.

Figure 3. ***Flowchart of the 'Healthy Toddler' Intervention***

**Steps according Persuasion-Communication Model (McGuire, 1985)**	**Element of the 'Healthy Toddler' intervention Package**	**Content / activities**	**Age of the child**
Attention for the health message	E-Health Module	- Assessment of four key behaviours<br>- Assessment of parenting behaviour<br>- Feedback on four key behaviours, in comparison with recommendations<br>- Tailored advise, taking into account the four behaviours and parenting practices	± 18 months
Changing determinants and behaviour:<br>- Attitude<br>- Self-efficacy<br>- Parenting behaviour	Counselling with motivational interviewing technique	- Making parents aware of their child's behaviour<br>- Motivate parents to prevent overweight in their child<br>- Stress the parents responsibility and importance strict parenting<br>- Motivate parents to change their parenting<br>- Discuss advantages of strict parenting<br>- Discuss potential barriers (e.g. time constraints)<br>- Improve self-efficacy<br>- Provide parents skills:<br>- Setting rules, giving good example, use praise and award, do not use food as a means of control	± 18 months
Maintain behaviour change	Reminder	Reminder of tailored advice from E-Health module<br>Positive feedback	± 18 months
	2nd E-health Module	- Assessment four key behaviours<br>- Assessment of parenting behaviour<br>If behaviour change was achieved, parents receive positive feedback<br>If not, parents receive tailored advice	± 24 months
	2nd Counselling with motivational interviewing technique	- Assessment of behaviour change<br>- if yes, provide positive feedback<br>- If not, repeat activities described in 1st counselling	± 24 months
	2nd Reminder	Reminder of tailored advice from E-Health module	± 24 months

6. METHODS

MEASUREMENTS AND OUTCOME MEASURES

Given informed consent, data will be collected from the YHC-files of the participating families/children; parent-questionnaires to be completed at home; registration forms to be completed by YHC-professionals at the end of distinct YHC-contact moments (mainly process information); brief assessment forms to be completed by parents after distinct YHC-contact moments (process information). Anthropometrical measures (length, weight; waist circumference from age 24 months on) will be recorded according to standardized YHC protocols at the YHC-centers.

T0. DATA COLLECTION AT BASELINE (CHILD AGE CIRCA 1 MONTH)

The following data will be collected at baseline (T0).

- Pregnancy and delivery data: gestation; complications during pregnancy; mode of delivery; weight gain during pregnancy;
- Newborn: gender; birth ranking; birth weight and length; lowest weight; breast/bottle feeding;
- Parents: age; self-reported length and weight; health status; occupation; educational level; country of birth/ethnicity; history of weight problems; history of eating disorders;
- Siblings: gender; birth ranking; age; length and weight from file or parent-report.

T1. DATA COLLECTION AT CHILD AGE 6 MONTHS

The following data will be collected at child age 6 months (T1).

- Weight and length of the child (from YHC-file);
- History of breast/bottle feeding; supplemental feeding/introduction of solid food; family eating practices;
- Health and well-being of the parent: SF-12 (Gandek et al., 1998);
- In case of siblings in the family: Parent Problem Checklist (PPC) (Dadds et al., 1991);
- Health and well-being of the child: subscales of the Infant Toddler Quality of Life questionnaire (ITQOL) (Raat et al., 2007).

T2. DATA COLLECTION AT CHILD AGE 15 MONTHS

The following data will be collected at child age 15 months (T2).

- Weight and length of the child (from YHC-file at age 14 months);
- History of breast/bottle feeding; supplemental feeding/introduction of solid food;

- Energy balance-related behaviors (‘BOFT’ behaviors): exercise and outdoor playing, having an appropriate family breakfast daily, sugar sweetened drinks consumption, and TV viewing, measured by parental questionnaires (Raat et al., 2007);
- Attitudes, motivation and perceived control of parents regarding the four energy balance-related (‘BOFT’) behaviors (Raat et al., 2007);
- Child rearing questionnaire for parents; Opvoedingsvragenlijst Voor Ouders (OVO) (Dekovic et al, 1991);
- Parenting style (PS) (Arnold et al., 1993);
- Parenting practices and rules (PP) (Arredondo et al, 2006);
- Health and well-being of the child: brief-Infant-Toddler Social and Emotional Assessment (brief ITSEA) (Briggs-Gowan et al., 2004).

TO BE REGISTERED IN YHC-CENTERS (24 MONTHS)

Additionally will be recorded at child age 24 months:

Weight and length of the child, and waist circumference.

T3. DATA COLLECTION AT CHILD AGE 36 MONTHS

The following data will be collected at child age 36 months (T3).

- Weight and length of the child, and additionally waist circumference (from YHC-file at age 36 months);
- Energy balance-related behaviors (‘BOFT’ behaviors): exercise and outdoor playing, having an appropriate family breakfast daily, sugar sweetened drinks consumption, and TV viewing, measured by parental questionnaires (Raat et al., 2007);
- Attitudes, motivation and perceived control of parents regarding the four energy balance-related (‘BOFT’) behaviors (Raat et al., 2007);
- Child rearing questionnaire for parents; Opvoedingsvragenlijst Voor Ouders (OVO) (Dekovic et al, 1991);
- Parenting style (PS) (Arnold et al., 1993);
- Parenting practices and rules (PP) (Arredondo et al, 2006);
- Parent Problem Checklist (PPC) (Dadds et al., 1991)
- Parents: self-reported length and weight;
- Siblings: length and weight from YHC-files (if available);
- Health and well-being of the parent: SF-12 (Gandek et al., 1998);
- Health and well-being of the child: subscales of the Infant Toddler Quality of Life questionnaire (ITQOL) (Raat et al., 2007);
- brief-Infant-Toddler Social and Emotional Assessment (brief ITSEA) (Briggs-Gowan et al., 2004).

The primary outcome measures:

** Energy balance-related behaviors: exercise and outdoor playing, having an appropriate family breakfast daily, sugar sweetened drinks consumption, and TV viewing, measured by parental questionnaires (Raat et al., 2007).

** BMI (weight/height2) according to standardized procedures during scheduled YHC visits; waist circumference (Eisenmann et al., 2004).

** Additionally: Proportion of overweight children based on internationally accepted gender and age-specific cut-off values (Cole et al., 2000)
.

The secondary outcome measures:

** Attitudes, motivation and perceived control of parents regarding the four energy balance-related ('BOFT') behaviors (Raat et al., 2007).

** General parenting styles and parental practices and rules. These will be measured by Child rearing questionnaire for parents (Opvoedingsvragenlijst Voor Ouders; OVO) (Dekovic et al, 1991); Parenting style (PS) (Arnold et al., 1993); Parenting practices and rules (PP) (Arredondo et al, 2006).

** Child's health and well-being: Infant Toddler Quality of Life questionnaire (ITQOL) (Raat et al., 2007); brief-Infant-Toddler Social and Emotional Assessment (ITSEA) (Briggs-Gowan et al., 2004).

Co-variates:

** Pregnancy characteristics of the child, birth weight and length, lowest weight.

** Parents' and siblings' height and weight using self report and medical files. Self-report of adult height and weight has proven to be sufficiently accurate for epidemiological research (McAdams et al., 2007).

** Parents' health and well-being (SF-12; Parent Problem Checklist).
** General demographics, including educational level and ethnicity of the parents.

** Non response measures, including general demographics and reasons for non-participation

Process measures:

In both intervention groups (‘BOFT+’ and the ‘Healthy toddler’) process characteristics of the interventions will be measured additionally by brief questionnaires for parents and for YHC professionals regarding each of the intervention elements, as developed in prior projects of the research group. Adherence to the distinct elements of the Prevention-program will be registered by the YHC-team. The process measures include:

** Adherence to the distinct elements of the intervention (YHC Professionals), using form completed directly after the contact.

** Appreciation/satisfaction regarding the interventions (YHC professionals and parents) with regard to the distinct elements. (Mangunkusumo et al., 2007).

** Description of additional time investment (and appreciation) by YHC-professionals and parents.

Measures regarding the Cost-effectiveness evaluation:

With regard to the costs of the ‘BOFT+’- and the ‘Healthy toddler’ intervention, we will assess the costs of additional time of YHC contacts, training of YHC-personnel, other program costs (e.g. material costs; non-client related time investments of YHC-personnel with respect to the intervention package). Data on time investments, expenditures and program costs will be collected by questionnaires among YHC-personnel and parents (see Process evaluation), and from YHC administrative data sources.

<u>PROCEDURE OF THE STUDY</u>

Training of the YHC professionals in the two intervention groups
Before the start of both interventions, the YHC professionals (generally the YHC-nurses and YHC-physicians) belonging to the 2 intervention groups, will be trained to apply the ‘BOFT+’ respectively the ‘Healthy toddler’ intervention.

Training regarding the ‘BOFT+’ intervention:
The team of the well-baby clinic usually consists of one doctor and a one or two Youth Health Care nurses. The 15 ‘BOFT+’ intervention teams will be trained before start of the study. The training consists of 2 sessions of 4 hours each. During the course of the project (from start of inclusion to the final intervention in the study will take circa 2 1/2 to 3 years), ‘booster’ sessions will be planned every six months to motivate the ‘BOFT+’ intervention teams and to ensure proper compliance to the protocol of the intervention. Every training will be focused on another age group, but exactly the same principles from the learning theory will be used.

Training regarding the ‘Healthy toddler’ intervention:
During a full-day session prior to the start of the ‘Healthy toddler’ intervention and a half-day ‘booster’ session 4 months after the start of the intervention in order to rehears the trained skills, YHC professionals will be trained in using the motivational interviewing in the context of the YHC-Overweight prevention protocol. This training will be given by a professional, certified trainer of motivational interviewing techniques as applied in (preventive) health care in cooperation with the Knowledge Center for Overweight (Kenniscentrum Overgewicht). Training will be in small groups including circa 10-15 YHC professionals.

According to the general principles of motivational interviewing, the following elements will be included in the training in order to enhance self-management by parents regarding their parenting style and parenting practices concerning improvement of the four energy balance-related behaviors:

Stimulation of making parents aware of the risk for overweight and its potential adverse effect for their child. Stimulation of favorable change of parental attitudes and motivations regarding overweight prevention and authoritative parenting. Stimulation of the parents perception of being responsible for the health of their child.Stimulation goal setting and providing feed-back in the context of realistic goals that may increase authoritative parenting and improve energy balance-related behaviors in their child. Providing information on beneficial parental practices and stimulating to teach parents these skills. Providing information in order to find specific information when parents have specific questions regarding parenting or overweight prevention.

Numbers of parents/children included in the study
A total of 45 YHC-teams of circa ten regional services providing Youth Health Care, will participate in the study. We assume, on average, 15 toddlers per age group per month per YHC-team to be scheduled for a regular visit at a certain age (i.e. 2 weeks after birth at the start of the inclusion procedure). In the course of an inclusion period of maximally 12 months in the project, the 45 participating YHC-teams together will have invited a total of 3.000 parents/toddlers in the study (two intervention groups and one control group).
Taking into account informed consent by 50% and dropout between baseline and follow-up of 30%, we expect complete data from a total of 1.050 parents/toddlers at follow-up, equally divided over the control group and the two intervention groups.

Information about the study, informed consent, invitation to participate, baseline measurements
All parents of the children in the study population will, circa 2 weeks after birth, during a regular home visit by the YHC-nurse be informed about the study and will be invited to participate; this can be discussed at and informed consent can be given at the first regular visit to the YHC-center at age of circa one month old. Parents receive information about the study; informed consent form; and a brief questionnaire with items on background data (see above; data gathering at T0).

Intervention group (a): 'BOFT+'

Parents that are allocated to a 'BOFT+' YHC-team will receive care during the regularly scheduled visits. However their YHC-professionals will be trained according to the 'BOFT+' principles and the content of child rearing advices will be influenced by those principles; at certain contact moments specific supporting materials will be offered to the parents (see above and see 'BOFT+' annex).

Intervention group (b): the 'Healthy toddler'

In case of the 'Healthy toddler' intervention group, parents will also receive care during the regularly scheduled visits. However, one month prior to the visit at child age 18 months, these parents will receive an invitation to visit the 'Healthy toddler' website to obtain tailored information prior to the YHC-visit. Parents can participate via Internet or by completing a paper assessment questionnaire that should be sent back by mail. Parents completing the Internet version will receive tailored feedback immediately. Parents completing the paper version will be mailed a paper version of the tailored advice.

Parents that are allocated to a 'Healthy toddler' intervention YHC-team will be offered a 18-month visit that is 15 minutes longer than a regular visit and that includes face-to-face counseling based on motivational interviewing. During the counseling the parents can discuss any issue that arose from the tailored feedback.

Circa one month after the face-to-face counseling, these parents will receive a reminder by email or by mailed letter, including the tailored advice.

At child age of circa 24 months, circa one month before the next regular visit to the YHC center, the parents in this group will again receive a request to complete the 'Healthy toddler' assessment by Internet or via paper, and receive tailored feedback. The feedback can be discussed during the YHC-visit that will be allowed 10 minutes extra time for face-to-face counseling based on motivational interviewing. Circa one month after the face-to-face counseling, these parents will receive a reminder by email or by mailed letter, including the tailored advice.

7. STATISTICAL ANALYSIS

Intervention effect:

To assess the intervention effect, multilevel analyses will be applied to allow for dependency between the individual measurements within the randomized YHC-teams (Campbell et al., 2004). The multilevel approach is also appropriate for the analyses regarding longitudinal data in the study. Multilevel linear regression analyses will be conducted for the continuous outcome variables with group (intervention or control) as independent variable and baseline values and potential confounders as covariates. Multilevel logistic regression will be performed in case of dichotomous outcome variables.
Additionally, effect modification by gender, parental educational level, ethnicity, and parental overweight status will be conducted by introducing an interaction term into the regression analyses. If this interaction term reaches statistical significance, stratified analyses will be carried out.

Mediation of the effect:

We will conduct a mediation analyses in order to verify whether parenting styles and practices can explain the achieved intervention effect regarding energy balance-related behaviors. Mediation of the intervention effect by important process measures will additionally be assessed (Mangunkusumo et al., 2007). Analyses will be conducted according suggestions by Baron et al. (1986) using multilevel regression analyses and structural equation modeling.

Descriptive analyses of process measures:

Adherence to and satisfaction with the distinct elements of the ‘BOFT+’ and the ‘Healthy toddler’ interventions by parents and professionals will be described. We will explore YHC-center characteristics and parent/toddler characteristics that are associated with the degree of adherence and satisfaction using multiple linear or logistic regression analyses.

Cost-effectiveness analyses:

Costs and effects will be evaluated for the purpose of a cost-effectiveness analysis. Potential future reduction in the prevalence of overweight will be estimated together with an estimation of costs saved (Haby et al., 2006).

8 ETHICAL CONSIDERATIONS

8.1 Regulation statement

The study will be conducted according to generally accepted guidelines of good practice.

8.2 Recruitment and consent

See the Procedure described above.

8.3 Objection by minors or incapacitated subjects (if applicable)

The study is aimed at parents who receive generally accepted advices regarding nutrition and playing time of their infants and toddlers; in this context the parenting by parents will be supported by YHC-professionals.

8.4 Benefits and risks assessment, group relatedness

In the three arms of the study generally accepted approaches will be applied by the YHC-professionals. There are no specific risks for the participants in the study.

8.5 Compensation for injury

According to our opinion no insurance is necessary.

8.6 Incentives (if applicable)

The participants in the study (the parents) will receive no incentives.

9 ADMINISTRATIVE ASPECTS AND PUBLICATION

9.1 Handling and storage of data and documents

Data will be handled confidentially and where possible anonymously. Where it is necessary to be able to trace data to an individual subject, a subject identification code list will be used to link the data to the subject. The code will not be based on the patient initials and birth-date. The key to the code will be safeguarded by the investigator. The handling of personal data will comply with the Dutch Personal Data Protection Act (in Dutch: De Wet Bescherming Persoonsgegevens, Wbp).

9.2 Amendments

All amendments will be notified to the METC that gave a favourable opinion.

9.3 Annual progress report

The investigator will submit a summary of the progress of the trial to the accredited METC once a year. Information will be provided on the date of inclusion of the first subject, numbers of subjects included and numbers of subjects that have completed the trial, serious adverse events/ serious adverse reactions, other problems, and amendments.

9.4 End of study report

The investigator will notify the accredited METC of the end of the study within a period of 8 weeks. The end of the study is defined as the last participant’s last measurement or visit.

In case the study is ended prematurely, the investigator will notify the accredited METC, including the reasons for the premature termination.

Within one year after the end of the study, the investigator/sponsor will submit a final study report with the results of the study, including any publications/abstracts of the study, to the accredited METC.

9.5 Public disclosure and publication policy

This study will be conducted with financial support of ZonMw, Prevention program. ZonMw promotes dissemination of all results of the study, without restrictions.

REFERENCES

Neppelenbroek, S.E., van Wijngaarden JCM, Lim-Feijen JF, van Leerdam F.J.M., Raat H, HiraSing R.A., Update Programmeringsstudie effectonderzoek jeugdgezondheidszorg 0-19 jaar. Utrecht: GGD Nederland; 2005.

Kremers, S.P.J., et al. *Parenting style and adolescent fruit consumption.* Appetite, 2003. **41**(1): p. 43-50.

te Velde SJ et al. Effects of a comprehensive fruit and vegetable promoting school-based intervention in three European countries: the Pro Children Study. British Journal of Nutrition 2007.

Van der Horst, K., et al., *Perceived parenting style and practices and the consumption of sugar-sweetened beverages by adolescents.* Health Educ. Res., 2006: p. cyl080.

De Bourdeaudhuij, I., et al., *Personal, social and environmental correlates of vegetable intake in normal weight and overweight 11-year old boys.* Int J Behav Nutr Phys Act, 2006. **3**(37).

van Wouwe JP, Mattiazzo GF, el Mokadem N, Reeser HM, Hirasing RA. The incidence and initial symptoms of diabetes mellitus type 1 in 0-14-year-olds in the Netherlands, 1996-1999. Ned Tijdschr Geneeskd 2004;148(37):1824-9.

Bosma H, Van de Mheen HD and Mackenbach JP. Social class in childhood and general health in adulthood: questionnaire study of contribution of psychological attributes. British Medical Journal 1999;318:18-22

Graafmans WC, Verloove-Vanhorick SP, Mackenbach JP et al. Birthweight and perinatal mortality; a comparison of optimal birthweight in seven western European countries. Epidemiology 2002;**13**(5): 569-74

Mackenbach JP, Kunst AE, Cavelaars AEJM, Groenhof F, Geurts JJM and the EU Working Group on Socioeconomic Inequalties in Health. Socioeconomic inequalities in morbidity and mortality in Western Europe. Lancet 1997;349:1655-1659

Brug J, van Lenthe F: Environmental determinants and interventions for physical activity, diet and smoking: A review. In. Rotterdam, The Netherlands: Erasmus MC; 2005.

Brug J, Oenema A, Kroeze W, Raat H. The internet and nutrition education: challenges and opportunities. European Journal of Clinical Nutrition 2005;59(Suppl 1):S130-S139.

Brug J, Oenema A, Campbell M. Past, present, and future of computer-tailored nutrition education. Am J Clin Nutr 2003;77(4 Suppl):1028S-1034S.

Oenema A, Brug J, Lechner L. Web-based tailored nutrition education: results of a randomized controlled trial. Health Educ Res 2001;16(6):647-60.

Oenema A, Brug J. Feedback strategies to raise awareness of personal dietary intake: results of a randomized controlled trial. Prev Med 2003;36(4):429-39.

Van Assema P, Brug J, Ronda G, Steenhuis I, Oenema A. A short dutch questionnaire to measure fruit and vegetable intake: relative validity among adults and adolescents. Nutr Health 2002;16(2):85-106.

Raat H, Bonsel GJ, Essink-Bot ML, Landgraf JM, Gemke RJ. Reliability and validity of comprehensive health status measures in children: The Child Health Questionnaire in relation to the Health Utilities Index. J Clin Epidemiol 2002;55(1):67-76.

Raat H, Bonsel GJ, Hoogeveen WC, Essink-Bot ML. Feasibility and reliability of a mailed questionnaire to obtain visual analogue scale valuations for health states defined by the Health Utilities Index Mark 3. Med Care 2004;42(1):13-8.

Raat H, Botterweck AM, Landgraf JM, Hoogeveen WC, Essink-Bot ML. Reliability and validity of the short form of the child health questionnaire for parents (CHQ-PF28) in large random school based and general population samples. J Epidemiol Community Health 2005;59(1):75-82.

Raat H, Bueving HJ, de Jongste JC, Grol MH, Juniper EF, van der Wouden JC. Responsiveness, longitudinal- and cross-sectional construct validity of the Pediatric Asthma Quality of Life Questionnaire (PAQLQ) in Dutch children with asthma. Qual Life Res 2005;14(1):265-72.

Mangunkusumo RT, Moorman PW, Van Den Berg-de Ruiter AE, Van Der Lei J, De Koning HJ, Raat H. Internet-administered adolescent health questionnaires compared with a paper version in a randomized study. J Adolesc Health 2005;36(1):70 e1-6.

Koomen I, Raat H, Jennekens-Schinkel A, Grobbee DE, Roord JJ, Van Furth AM. Academic and behavioral limitations and health-related quality of life in school-age survivors of bacterial meningitis. Quality of Life Research 2005;14:1563-1572.

Raat, H., et al., Reliability and validity of the Infant and Toddler Quality of Life Questionnaire (ITQOL) in a general population and respiratory disease sample. Qual Life Res 16(3): 445-60.

Mangunkusumo, R., Brug, J., Duisterhout, J., de Koning, H., Raat, H, *Feasibility, acceptability, and quality of Internet-administered adolescent health promotion in a preventive-care setting.* Health Educ Res, 2007. **22**(1): p. 1-13.

Sleuwen BE van, L'Hoir MP, Engelberts AC, Westers P, Schulpen TWJ. Infant care practices related to cot death in Turkish and Moroccan families in the Netherlands. Arch Dis Child 2003;88:784-8

Mitchell EA, Blair P, L'Hoir MP. Should pacifiers be recommended to prevent Sudden Infant Death Syndrome? Pediatrics 2006;117:1755-8

Gestel JPJ van, L'Hoir MP, Berge M ten, Jansen NJG, Plötz FB. Risks of ancient practices in modern times. Pediatrics 2002;110:e78

Sleuwen BE van, L'Hoir MP, Engelberts AC, Busschers WB, Westers P, Blom MA, Schulpen TWJ, Kuis W. Comparison of behaviour modification with and without swaddling as interventions for excessive crying. J Pediatr 2006;149:512-7

Sleuwen BE van, Engelberts AC, Boere-Boonekamp MM, Kuis W, Schulpen TWJ, L'Hoir MP. Swaddling – a systematic review. Pediatrics 2006

Sleuwen EA, L'Hoir MP, Engelberts AC, Busschers WBB, Westers P, Blom MA, Schulpen TWJ, Kuis W. Swaddling, an effective intervention in excessive crying? J of Pediatrics. jpeds.2006.06.068:512-7

Sleuwen BE van, Engelberts AC, Boere-Boonekamp MM, L'Hoir MP. Behaviour modification and swaddling as interventions to improve sleep: A link with obesity? Arch Dis Child jan 3rd 2007 (electronic pages)

L'Hoir M.P., Engelberts A.C., Well van G.Th.J., Westers P., Mellenbergh G.J., Wolters W.H.G., Huber J. Case-control study of current validity of previously described risk factors for SIDS in the Netherlands. Arch Dis Child 1998; 1998: 0-7

Vlimmeren LA, Graaf Y van der, Boere-Boonekamp MM, L'Hoir MP, Helders PJM, Engelbert RHH. Risk factors for deformational plagiocephaly at birth and at seven weeks of age - A prospective cohort study. Pediatrics 2007;119;408-18

L'Hoir MP, Boere-Boonekamp MM, Beltman M, Bruil J, Dijkstra N, Engelberts AC. Preventie van overgewicht bij niet-westerse zuigelingen. Tijdschr Jeugdgezondheidszorg 2006;5:91-4

Boere-Boonekamp MM, Kerkhoff AHM, Schuil PB, Zielhuis GB. Early Detection of Developmental Dysplasia of the Hip in the Netherlands: The Validity of a Standardized Assessment Protocol in Infants. Am J Public Health 1998;88:285-8

Boere-Boonekamp MM, Linden-Kuiper AT van der. Positional Preference: Prevalence in Infants and Follow-Up After Two Years. Pediatrics 2001;107:339-43

Boere-Boonekamp MM, Haasnoot-Smallegange ME, Rutten AL Borstvoeding en groei: kennis, attitude en gedrag van medewerkers jeugdgezondheidszorg. Tijdschr Jeugdgezondheidszorg 2005;2:30-4

Roovers EA, Boere-Boonekamp MM, Castelein RM, Zielhuis GA, Kerkhoff AHM. Effectiveness of ultrasound screening for developmental dysplasia of the hip. Arch Dis Child Fetal Neonatal Ed 2005;90:F25-F30

Blokland G, Prinsen B, Kok C, Wijngaarden J van. De Jeugd heeft de toekomst. Preventie van psychosociale problematiek bij jeugdigen, maatwerk van de GGD. Utrecht, NIZW 2003

+ + + + + + + + + +

Ajzen I (1986) Prediction of goal-directed behavior: Attitudes, intentions, and perceived behavioral control. Journal of Experimental Social Psychology 22, 453-474.

Atkin LM et al, Diet composition and body composition in preschool children. Am J Clin Nutr 2000, 72: 15-21.

Bandura A (1986) Social Foundations for Thought and Action: a Social Cognitive Theory. Englewood Cliffs, NJ: Prentice Hall.
Barlow J et al., Preventing emotional and behavioral problems: the effectiveness of parenting programmes with children less than 3 years of age. Child Care Health Dev 2005;31:33-42

Baron RM et al, The moderator-mediator variable distinction in social psychological research. Journal of Personality and Social Psychology 1986, 51(6):1173-1182.

Basistakenpakket Jeugdgezondheidszorg 0-19 jaar. Ministerie van Volksgezondheid Welzijn en Sport; 2002.

Bertrand R. et al, Behoefte aan opvoedingsondersteuning in Nederlandse, Marokkaanse en Turkse gezinnen met kinderen van 0-6 jaar. Nederlands Tijdschrift voor Opvoeding, Vorming en Onderwijs 1998;14: 50-71

Brug J van et al, Environmental determinants and interventions for physical activity, nutrition and smoking: a review. Erasmus MC Rotterdam; 2005.

Bulk-Bunschoten AMW et al, Signaleringsprotocol overgewicht in de jeugdgezondheidszorg. Woerden: Platform Jeugdgezondheidszorg; 2005.

Campbell MK et al, CONSORT statement: extension to cluster randomised trials. BMJ 2004, 328(7441):702-708.

Cole TJ et al, Establishing a standard definition for child overweight and obesity worldwide: international survey. BMJ 2000, 320(7244):1240-3.

Darling N et al (1993) Parenting Style as Context - an Integrative Model. Psychological Bulletin 113, 487-496.

Dekovic M et al, Factor structure and construct validity of the Block Child Rearing Practices Report (CRPR). Psychological Assessment 1991;3:182-7.

Eisenmann JC et al, Assessing body composition among 3- to 8-year-old children: anthropometry, BIA, and DXA. Obes Res 2004, 12(10):1633-1640.

Exter Blokland EAW den et al, Parenting styles, self-control and male juvenile delinquency: the mediation role of self-control. In: Prevention and Control of Aggression and the Impacts on Its Victims. Edited by Martinez M. Dordrecht, the Netherlands: Kluwer; 2001: 201-207.

Fredriks AM et al, Continuing positive secular growth change in The Netherlands 1955-97. Pediatric Research 2000;47:316-23

Fredriks AM et al, Alarming prevalences of overweight and obesity for children of Turkish, Moroccan and Dutch origin in The Netherlands according to international standards. Acta Paediatr 2005, 94(4):496-498.

Golan M et al (2006) Childhood obesity treatment: targeting parents exclusively v. parents and children. Br J Nutr 95, 1008-1015.

Green LW et al (1996) Ecological foundations of health promotion. Am J Health Promot 10, 270-281.

Haby MM et al (2006) A new approach to assessing the health benefit from obesity interventions in children and adolescents: the assessing cost-effectiveness in obesity project. 30, 1463-1475.

Hirasing RA et al, Het overbruggingsplan voor kinderen met overgewicht. Amsterdam: VU medisch centrum; 2005.

Holmbeck GN (1997) Toward terminological, conceptual, and statistical clarity in the study of mediators and moderators: examples from the child-clinical and pediatric psychology literatures. J Consult Clin Psychol 65, 599-610.

Korfage, I.J. et al (2002) Tijdbesteding en kosten van het consultatiebureau voor ouder- en kindzorg. TSG 80, 436-441.

Kremers SPJ et al (2003) Parenting style and adolescent fruit consumption. Appetite 41, 43-50.

Laar CWF van de et al, Preventie van overgewicht: een minimale interventie strategie bij 5/6 jarige kinderen binnen de JGZ. NWO-werkgemeentschap Jeugd en Gezondheid: 2006.

Lamborn SD et al (1991) Patterns of competence and adjustment among adolescents from authoritative, authoritarian, indulgent, and neglectful families. Child Dev 62, 1049-1065.

L'Hoir MP, Boere-Boonekamp MM et al, Preventie van overgewicht bij niet-westerse zuigelingen. Tijdschr Jeugdgezondheidsz 2006;5:91-4

McAdams MA et al (2007) Comparison of Self-reported and Measured BMI as Correlates of Disease Markers in U.S. Adults. Obesity (Silver Spring) 15, 188-196.

McGuire WJ (1985) Attitudes and attitude change. In The handbook of social psychology, pp. 233-346 [G Lindzey and E Aronson, editors]. New York: Random House.

Ministerie van VWS. Preventienota ´Langer gezond leven 2004-2007, ook een kwestie van gezond gedrag´. Den Haag, VWS 2003

Nader PR et al, Identifying Risk for Obesity in Early Childhood. Pediatrics 2006;118;594-601

O'Connor TM, Yang S-J & Nicklas TA (2006) Beverage Intake Among Preschool Children and Its Effect on Weight Status. Pediatrics 118, e1010-1018.

Parsons TJ et al, Childhood predictors of adult obesity: a systematic review. Int J Obesity 1999;23(suppl 8):S1-107

Renders CM et al, Kinderen met overgewicht en obesitas en preventieve maatregelen. Ned Tijdschr Geneeskd. 2004;148:2066-70

Renders CM, et al, Televisiekijken en enkele eetgewoonten bij Amsterdamse 6-14-jarigen; een transversaal onderzoek. Ned Tijdschr Geneeskd 2004, 148(42):2072-2076.

Rhee KE et al, Parenting Styles and Overweight Status in First Grade. Pediatrics 2006;117;2047-54

Rhee KE et al, Factors associated with parental readiness to make changes for overweight in children. Pediatrics 2005;116:e94-101

Robinson TN: Reducing children's television viewing to prevent obesity: a randomized controlled trial. Jama 1999, 282(16):1561-1567.

Rollnick S et al, Consultations about changing behaviour. BMJ 2005, 331(7522):961-963.

Sanders MR et al (2003) Theoretical, scientific and clinical foundations of the Triple P-Positive Parenting Program: a population approach to the promotion of parenting competence. Parenting research and practice monograph 1, 1-24.

Schregardus RC. Kinderen met slaapproblemen. Amsterdam: Boom, 1993

Sessa FM (1992) Family structure, parenting, and adolescent adjustement, Temple University.

Stam PCC van. Invloed van opvoedingsstijl op het ontstaan van overgewicht bij kinderen. Een literatuuroverzicht. Utrecht: Universiteit Utrecht, 2005

Steinberg L et al (1989) Authoritative parenting, psychosocial maturity, and academic success among adolescents. Child Dev 60, 1424-1436.

Summerbell CD et al, Interventions for treating obesity in children. Cochrane Database Syst Rev 2003;(3):CD001872.

Van der Horst K et al (2006) Perceived parenting style and practices and the consumption of sugar-sweetened beverages by adolescents. Health Educ. Res., cyl080.

Whitlock EP et al, Screening and interventions for childhood overweight: a summary of evidence for the US Preventive Services Task Force. Pediatrics 2005, 116(1):e125-144.
